# Development of Novel Pyrrole Derivatives and Their Cinnamic Hybrids as Dual COX-2/LOX Inhibitors

**DOI:** 10.3390/molecules28247958

**Published:** 2023-12-05

**Authors:** Viola Noti, Eleni Pontiki, Dimitra Hadjipavlou-Litina

**Affiliations:** Department of Pharmaceutical Chemistry, School of Pharmacy, Faculty of Health Sciences, Aristotle University of Thessaloniki, 54124 Thessaloniki, Greece; notiviola@pharm.auth.gr (V.N.); epontiki@pharm.auth.gr (E.P.)

**Keywords:** inflammation, hybrids, pyrroles, cinnamic acid, multi-target, lipoxygenase inhibitor, anti-cyclooxygenase, antioxidant

## Abstract

Molecular hybridization has emerged as a promising approach in the treatment of diseases exhibiting multifactorial etiology. With regard to this, dual cyclooxygenase-2/lipoxygenase (COX-2/LOX) inhibitors could be considered a safe alternative to traditional non-steroidal anti-inflammatory drugs (tNSAIDs) and selective COX-2 inhibitors (coxibs) for the treatment of inflammatory conditions. Taking this into account, six novel pyrrole derivatives and pyrrole–cinnamate hybrids were developed as potential COX-2 and soybean LOX (sLOX) inhibitors with antioxidant activity. In silico calculations were performed to predict their ADMET (absorption, distribution, metabolism, excretion, toxicity) properties and drug-likeness, while lipophilicity was experimentally determined as R_M_ values. All synthesized compounds (**1**–**4**, **5**–**8**) could be described as drug-like. The results from the docking studies on COX-2 were in accordance with the in vitro studies. According to molecular docking studies on soybean LOX, the compounds displayed allosteric interactions with the enzyme. Pyrrole **2** appeared to be the most potent s-LOX inhibitor (IC_50_ = 7.5 μM). Hybrids **5** and **6** presented a promising combination of in vitro LOX (IC_50_ for **5** = 30 μM, IC_50_ for **6** = 27.5 μM) and COX-2 (IC_50_ for **5** = 0.55 μM, IC_50_ for **6** = 7.0 μM) inhibitory activities, and therefore could be used as the lead compounds for the synthesis of more effective multi-target agents.

## 1. Introduction

Inflammation is a natural defense mechanism that evolved in higher organisms to protect them against foreign and harmful stimuli. An inflammatory response can be either acute or chronic. The former is a beneficial process designed to protect the host organism from pathogens, damaged cells and toxic compounds. On the other hand, prolonged and uncontrolled reaction to inflammatory stimulus has been associated with the development and progression of several multifactorial conditions [1], such as obesity [2], diabetes [3], cardiovascular diseases [4], arthritis and joint diseases [5], allergies [6], chronic obstructive pulmonary disease (COPD) [7], neurodegenerative diseases [8] and various types of cancer [9].

Prostaglandins (PGs) from the cyclooxygenase (COX) pathway and leukotrienes (LTs) from the lipoxygenase (LOX) pathway are both pro-inflammatory mediators generated by the arachidonic acid (AA) cascade, and they have been implicated in the pathophysiology of chronic inflammatory conditions. Traditional non-steroidal anti-inflammatory drugs (tNSAIDs) are the most used medicines for the treatment of fever, pain and inflammation. Their main mechanism of action involves the non-selective inhibition of cyclooxygenase-1 (COX-1) and cyclooxygenase-2 (COX-2). These medications are associated with several adverse effects, including gastrointestinal toxicity due to COX-1 inhibition [10] and cardiovascular effects [11]. Moreover, patients of advanced age face a high risk of developing nephrotoxicity [12]. Coxibs, a group of highly selective COX-2 inhibitors, were developed in order to improve the gastrointestinal safety of anti-inflammatory therapy. Nevertheless, these agents increase the risk of cardiovascular adverse events [13], while their prolonged use upregulates the LOX activity, leading to elevated levels of leukotrienes that ultimately result in bronchoconstriction and increased airway inflammation [14].

**Figure 1:**
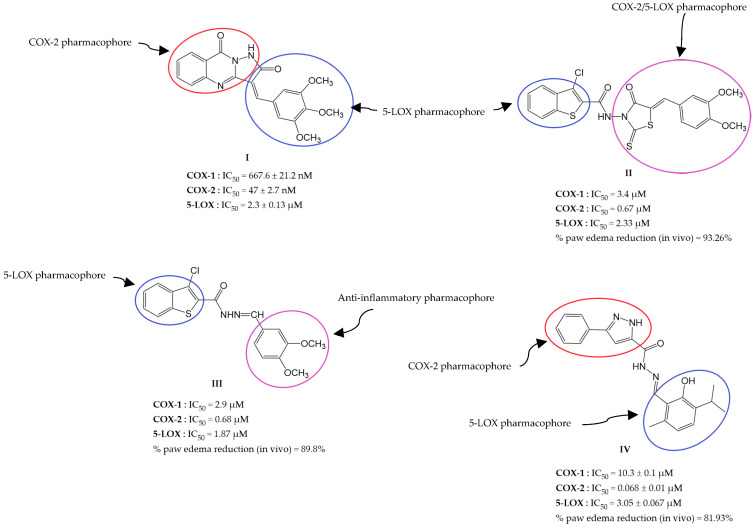
Structures and biological activities of dual COX-2/5-LOX inhibitors (**I**–**IV**) [16,17,18,19].

There is growing evidence that the selective inhibition of either the COX-2 or LOX pathway could shift the AA metabolism towards the other biosynthetic pathway leading to undesirable side effects [15]. Hence, there is an urgent need for new anti-inflammatory agents preventing the release of both prostaglandins and leukotrienes. Taking this into consideration, molecular hybridization is currently the most promising therapeutic approach. This strategy is based on the combination of the pharmacophoric units of different bioactive compounds, namely COX-2 and LOX inhibitors, to produce a new single chemical entity with synergistic effects and a better safety profile. Four representative hybrids (**I**–**IV**) given in the literature presenting with a dual mechanism of action are depicted in Figure 1 [16,17,18,19].

Over the years, accumulating data have highlighted the association between oxidative stress and inflammation. Oxidative stress is viewed as an imbalance between the generation of reactive oxygen species (ROS) and the availability of endogenous antioxidants. The overactivation of the COX-1/2 and LOX pathways during a chronic inflammatory response contributes to the excessive production of ROS, which could ultimately result in the oxidative damage of lipids, proteins, and nucleic acids [20,21].

**Figure 2:**
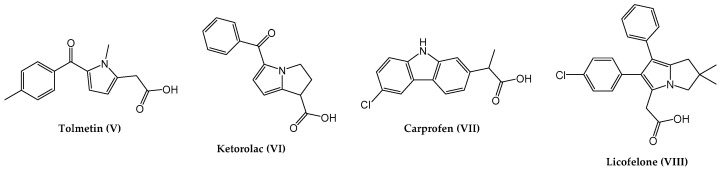
Structures of pyrrole-based anti-inflammatory compounds (**V**–**VIII**).

Pyrrole analogues represent one of the most important classes of heterocyclic compounds in medicinal chemistry. Among well-known NSAIDs, pyrrole derivatives such as tolmetin (**V**) [22], ketorolac (**VI**) [23], and carprofen (**VII**) [24] are of remarkable interest. Licofelone (**VIII**), a dihydropyrrolizine analogue (Figure 2), is a 5-LOX and a non-selective COX inhibitor that has successfully completed phase III trials for the treatment of osteoarthritis (OA) [25]. Furthermore, in the literature, there have been reports of 3,4-disubstitued-pyrrole derivatives inhibiting COX-1/COX-2, while presenting low or no action against LOX [26,27].

Cinnamic acid and its derivatives comprise a significant class of aromatic compounds with a broad range of biological applications. Several studies have reported the attractive multifunctional activities of cinnamic acid and its potential use as a multi-target agent. In addition to being used as the building block of other bioactive compounds, cinnamic acid exhibits antioxidant [28,29] and anti-lipoxygenase (anti-LOX) [30] activities.

In light of the above and in continuation of our previous study [31], our research was focused on the integration of the pyrrole and cinnamate moiety in the same molecule to obtain multi-target compounds. Chalcones were used as intermediates for the synthesis of pyrroles. These α,β-unsaturated carbonyl compounds are known for their structural diversity, ease of synthesis, and wide range of pharmaceutical applications [32], including their antioxidant [33] and anti-inflammatory properties [34,35].

**Scheme 1:**
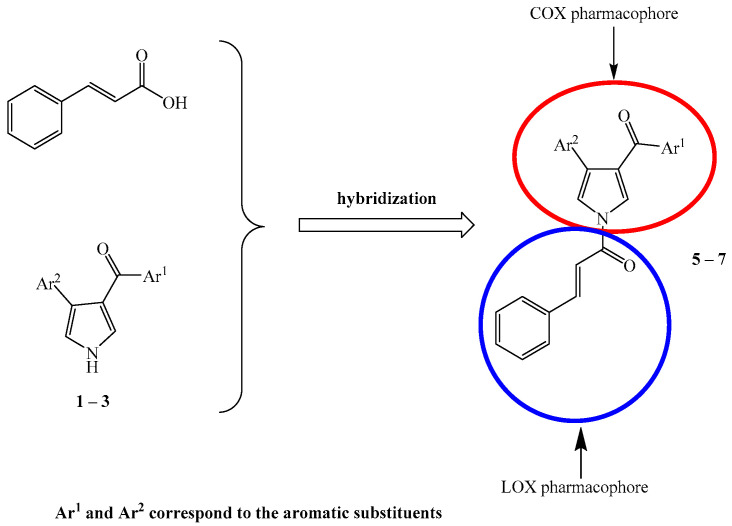
General structure of the synthesized pyrrole–cinnamate hybrids.

Herein, we present the design, in silico, of the synthesis, biological evaluation, and modelling studies of novel 3,4-disubstituted-pyrrole derivatives and pyrrole–cinnamic acid hybrids as potential dual COX-2/LOX inhibitors and antioxidant agents (Scheme 1).

In this regard, a library of novel pyrrolyl and pyrrole–cinnamate hybrid molecules was designed and screened utilizing a variety of useful in silico tools. For our previously published compounds **4** and **8**, the synthesis, biological activities, and the preliminary in silico study was performed and discussed in ref. [31]. Herein, an extended in silico ADMET study will be given for all the molecules and all the taken data will be fully discussed.

## 2. Results and Discussion

### 2.1. Chemistry

**Scheme 2:**
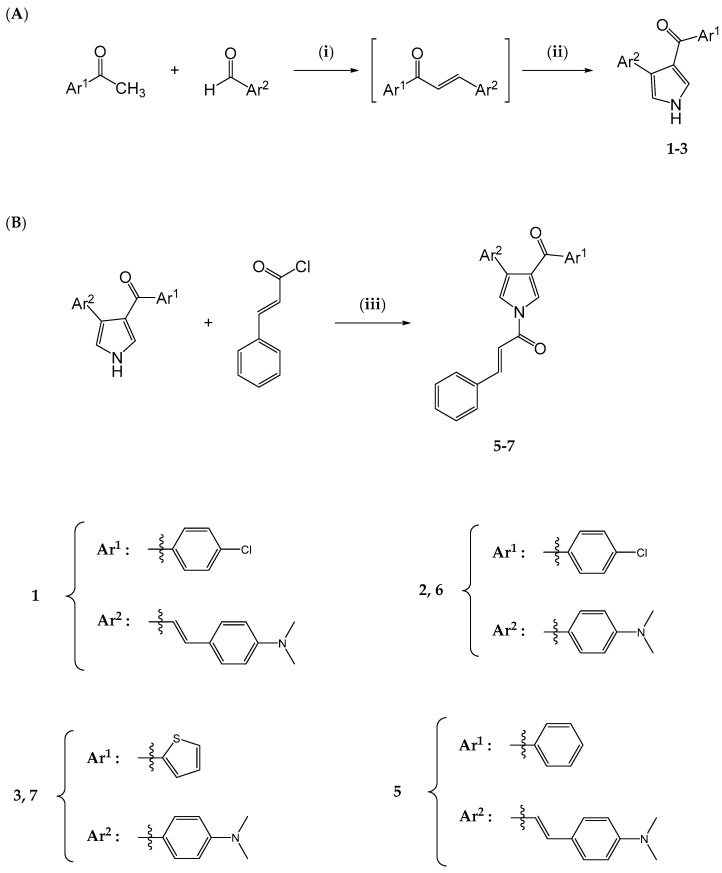
(**A**) One-pot synthesis of pyrrole analogues **1**–**3**. (**i**) LiOH.H_2_O (0.1 eq), EtOH absolute (3 mL), rt. (**ii**) LiOH.H_2_O (2.2 eq), *p*-tosylmethyl isocyanide (TosMIC) (2.4 eq), rt. (**B**) Synthesis of pyrrole–cinnamate hybrids **5**–**7**. (**iii**) Et_3_N (0.7 eq), 4-(dimethylamino) pyridine (DMAP) (0.1 eq), dry CH_2_Cl_2_ (4 mL), argon gas, rt.

The synthesized derivatives have been selected after our in silico analysis according to the ADMET results, the modeling studies, and the 2D-QSAR studies. Thus, we did not select to synthesize the hybrid of pyrrolyl **1**, since, according to our in silico results, this was not a satisfactory case. The synthesis of pyrrole analogues **1**–**3** and pyrrole–cinnamate hybrids **5**–**7** was carried out according to our previously described procedure [31] with minor modifications. Pyrrole derivatives were obtained via a one-pot reaction as depicted in Scheme 2. The first step involved a Claisen–Schmidt condensation between an aromatic enolizable ketone and a suitable aromatic aldehyde in the presence of a catalytic amount of lithium hydroxide monohydrate (LiOH.H_2_O) in absolute ethanol (EtOH) to give the intermediate chalcones. In the second step the intermediate chalcones reacted with an excess amount of *p*-tosylmethyl isocyanide (TosMIC) under basic conditions to produce the desired pyrrolyl derivatives **1**–**3** in low yields (18–39%). The hybridization of the isolated pyrroles was accomplished through the addition of cinnamoyl chloride in the presence of 4-(dimethylamino) pyridine (DMAP) as a catalyst and the auxiliary base triethylamine (Et_3_N), in dry dichloromethane (CH_2_Cl_2_), and under argon atmosphere, in low yields (25–37%).

The final products’ structures were verified by IR, ^1^H-NMR, ^13^C-NMR, LC-MS, and elemental analysis. Pyrroles **1**–**3** presented the characteristic absorbance of N-H (3182–3272 cm^−1^) and C=O (1603–1611 cm^−1^) groups, whereas the IR spectra of hybrids **5**–**7** displayed the characteristic peaks of the carbonyl groups attached to the amide bond (1693–1698 cm^−1^) and to the aryl ring (1615–1624 cm^−1^). The proton of the NH group of pyrrole derivatives appeared as a broad peak at 11.50–11.62 ppm. The alkenyl hydrogens of **1** were observed at 7.41 and 6.87 ppm with a *J*-coupling value of 16.6 Hz, which is indicative of *trans*-isomerism. For all hybrids **5**–**7**, cinnamic moiety’s double-bond protons appeared as two doublets at 8.06–8.07 ppm and 7.14–7.17 ppm, with a *J*-coupling value of 15.4 Hz confirming the trans stereochemistry. A survey of the ^13^C-NMR spectra revealed that the peak at 163 ppm corresponds to the amidic carbonyl carbon, while the chemical shift for the carbonyl carbon at position-3 appeared at 183–192 ppm. LC-MS and elemental analysis supported the proposed structures of the synthesized compounds.

### 2.2. Physicochemical Studies

#### 2.2.1. Experimental Determination of Lipophilicity as R_M_ Values

Lipophilicity is one of the most studied physicochemical properties in the drug discovery process and is frequently employed in structure–activity relationship (SAR) studies. It contributes to the ADMET (absorption, distribution, metabolism, excretion, toxicity) properties, solubility, permeability to the potency and efficacy of drugs [36,37]. Hence, we attempted to experimentally determine the lipophilicity as R_M_ values applying the reversed-phase thin layer chromatography (RPTLC) method. In TLC, the retention parameter, R_F_, is defined as the ratio between the migration distances of the compound and of the mobile phase, both measured from the sample application position. The term R_M_ was introduced to facilitate a linear relationship with the log P values, and it is defined as similar to the capacity factor, k, in HPLC.

Additionally, the logarithm of the partition coefficient was theoretically calculated by the Bio-Loom program of BioByte Corp [38] as ClogP values and by the Molinspiration software 2016.03 [39] as miLogP values. According to the experimental R_M_ values, the most lipophilic pyrrole derivatives were **7** (0.6488) and **1** (0.3076), an observation supported by both ClogP and miLogP values. Compounds **5** (−0.4174), **6** (−0.4771), and **7** (−0.4771) presented very low experimental lipophilicity values (Table 1).

The observed discrepancy between the experimental and calculated log P values could be attributed, apart from the recognized fact that every molecule is unique in its geometry and charge distribution, so that average calculations based on statistical data are not always expected to be accurate, to a possible formation of intra- or inter-molecular hydrogen bonds which mask polar groups.

#### 2.2.2. In Silico Determination of ADMET Properties and Drug-Likeness

The in silico drug-likeness and ADMET parameters of the synthesized compounds are presented in Supplementary Table S1.

The drug discovery and development process can be extremely challenging, costly, and time-consuming, with a high failure rate in clinical trials, mostly due to poor pharmacokinetics, lack of clinical efficacy, and high toxicity [40]. The in silico prediction of ADMET parameters and drug-likeness at an early stage has been widely used amongst academia and pharma industry to rule out weak drug candidates.

The cheminformatics software tools of Molinspiration (www.molinspiration.com) (accessed on 1 September 2021) [39] were used to calculate certain physicochemical parameters necessary for the prediction of Lipinski’s rule of five. Lazar toxicity predictions by in silico toxicology gmbh were employed to predict acute toxicity in certain aquatic organisms, as well as mutagenicity and carcinogenicity [41]. PreADMET (https://preadmet.webservice.bmdrc.org/) (accessed on 1 September 2021) [42] is a web-based application used to predict toxicity, ADME properties, and drug-likeness.

One of the most widely used approaches for evaluating drug-likeness is the rule of five developed by Lipinski et al. [43]. It is a rule of thumb determining the likelihood of a bioactive compound being an orally active drug in humans. Other similar sets of guidelines for the prediction of drug-likeness are CMC-like [44], Leadlike [45], MDDR-like [46], and WDI-like [47] rules. According to the rule of five, all synthesized compounds (**1**–**4**, **5**–**8**) could be described as drug-like.

Since the oral bioavailability of drugs is closely related to their intestinal membrane permeation, Caco2, and MDCK cell permeability as well as human intestinal absorption were predicted by PreADMET [42]. The synthesized molecules (**1**–**4**, **5**–**8)** were determined to be well absorbed through the human intestine and presented moderate permeability through Caco2 cell line. Compounds **4** and **8** showed moderate MDCK permeability, whereas derivatives **1**–**3** and **5**–**7** displayed low permeability.

Another factor that should be considered in the drug design is the blood–brain barrier (BBB) penetration. For drugs not acting at the central nervous system (CNS), passage through the BBB may lead to undesirable side effects. Herein, the degree of BBB penetration was measured using the brain-to-plasma concentration ratio predicted by PreADMET [42] and the logarithm of this ratio (logBB) calculated by Clark’s modified Equation (1) [48].

logBB = 0.152ClogP − 0.0148TPSA + 0.139,
(1)

where ClogP is the logarithm of the partition coefficient and TPSA is the topological polar surface area derived by the Molinspiration software [39]. Compounds with logBB > 0.3 cross the BBB readily, whereas those with logBB < −1.0 are poorly distributed through the BBB [48]. In this case, all hybrids, and pyrroles **1**, **2** appear to cross the BBB readily. According to PreADMET predictions, pyrroles showed high while hybrids presented moderate absorption to CNS.

Phase I metabolism is primarily catalyzed by the cytochrome P450 (CYP450) enzymes. Compounds acting as inhibitors, inducers, or substrates for a specific CYP enzymatic pathway could affect the metabolism of co-administered drugs. Thus, the in silico prediction of metabolism is essential for assessing the risk of drug–drug interactions early in the drug discovery process. CYP2C19, CYP2C9, CYP2D6, and CYP3A4 isoenzymes participate in the metabolism of many commonly prescribed drugs. Hence, their interactions with the compounds library were evaluated, employing the PreADMET application [42]. It is worth noting that none of the compounds acted as an inhibitor or substrate for CYP2D6.

P-glycoprotein is a transmembrane efflux pump that utilizes ATP to actively transport substances out of the cell [49]. Inducers of p-glycoprotein can reduce the bioavailability of other drugs, whereas inhibitors of p-glycoprotein increase the bioavailability of the transporter’s substrates. According to PreADMET [42], compounds **1**–**3** and **5**–**7** inhibit p-glycoprotein.

Generally, a high plasma protein binding limits the distribution of xenobiotics to the tissues in the body where they could be metabolized or interact with a pharmacological target. As a result, xenobiotics’ half-life is extended, the duration of activity is increased, and competitive displacement interactions may occur when other medications with plasma protein affinity are co-administered, leading to drug–drug interactions. With the exception of **3**, all the other final products were strongly bound to plasma proteins.

With regard to the toxicity profile of compounds, carcinogenicity in mice and rats, mutagenicity using the Ames test, and the ability to inhibit the human ether-ὰ-go-go-related gene (hERG) cardiac potassium channel, which is associated with cardiotoxicity, were predicted in silico by PreADMET [42]. All compounds were tested in silico against four strains of *Salmonella typhimurium* in the Ames test and did not show mutagenic activity in at least one strain. Pyrroles **1**–**4** were not carcinogenic in mice, while hybrids **5** and **8** did not show carcinogenicity in rats. The risk of inhibiting the hERG channel appeared to be medium for all compounds.

In addition, the previously published quantitative structure–activity relationships (QSARs) of our group [50] were applied to the compounds of the library in relation to the ADMET results and helped us pick the most promising structures.

### 2.3. Biological Evaluation

In the present study, the new 3,4-disubstituted pyrrole derivatives and pyrrole–cinnamate hybrids were evaluated in vitro for their antioxidant activity and their ability to inhibit soybean lipoxygenase and cyclooxygenase-2, in comparison to the well-known reference compounds trolox, nordihydroguaiaretic acid (NDGA) [30], and indomethacin [51]. For the sake of comparison, the previously published results of **4**, **8** [31], **9** [27], and cinnamic acid’s biological data [30] are also included (Table 2) The biological results are discussed in relation to the physicochemical properties of the molecules, e.g., the molecular volume (MV) and molar refractivity (MR) of the aromatic substituents Ar^1^ and Ar^2^ and ClogP and R_M_ values.

It is widely known that oxidative stress can induce lipid peroxidation. One of the major end products of the latter process are lipid-derived aldehydes, such as malondialdehyde (MDA), which is more chemically stable and membrane-permeable than ROS. These lipid aldehydes can participate in cross-linking with DNA and proteins and subsequently facilitate the development of various pathological states [52]. Herein, we used the water-soluble 2,2′-azobis(2-methylpropionamidine) dihydrochloride (AAPH) as a free radical initiator for the in vitro antioxidant assay due to its ability to generate alkylperoxy radicals in the presence of oxygen molecules at 37 °C [53]. In general, compounds **1**–**4**, **2**–**8** presented moderate or relatively low antioxidant activity and were less potent than cinnamic acid and the reference molecule trolox. Compounds **7** and **3** that have thienyl group as the substituent Ar^1^ showed the lowest anti-lipid peroxidation activity and no action at all, respectively (Table 2). Derivative **4** was the most potent pyrrole (44% at 100 μM), while **6** presented the highest antioxidant activity among all compounds (62% at 100 μM). Pyrroles **1** and **2** with high lipophilicity (ClogP of **1** = 5.7, ClogP of **2** = 5.05; R_M_ of **1** = 0.3076, R_M_ of **2** = 0.0116) and molecular volume (MV of **1** = 318, MV of **2** = 290) values appeared to be weak antioxidants (34% and 33% at 100 μM, respectively). The increase in lipophilicity as R_M_ values reduces the antioxidant activity of pyrrole–cinnamate hybrids, whereas the molecular volume does not seem to have any effect. The replacement of the thienyl ring (MR = 2.395) with a bulkier substituent such as the 4-chlorophenyl group (MR = 3.077) increased the antioxidant activity of pyrrole **2** (33% at 100 μM) and hybrid **6** (62% at 100 μM) in comparison to the inactive derivative **3** and the weak antioxidant **7** (31% at 100 μM), respectively. The presence of a bulkier substituent Ar^2^ decreased the anti-lipid peroxidation activity of **5** (36% at 100 μM) compared to **8** (58% at 100 μM). All hybrids exhibited stronger antioxidant activity than the corresponding pyrroles.

In vitro anti-LOX activities were measured against soybean lipoxygenase isoenzyme-1 (SLOX-1), which is widely used as a model for mammalian LOXs. SLOX-1 exhibits maximal activity at pH 9.0 and preferentially catalyzes the deoxygenation of the substrate to the 13-hydroperoxide derivative. The production of the latter was detected by its absorbance at 234 nm. All compounds were less potent than the reference molecule NDGA. Pyrrole **9** was inactive under the reported experimental conditions, while **3** and **4** displayed moderate anti-LOX activity (43% at 100 μM and IC_50_ = 100 μM, respectively). The most active pyrroles were **2** and **1** with IC_50_ values of 7.5 μM and 51.5 μM, respectively. All hybrids exhibited strong activity against soybean LOX. The most potent one was **6** with an IC_50_ value of 27.5 μM. The increase in the molecular volume and lipophilicity as ClogP values enhances the anti-LOX activity of hybrids. It seems that the increase in the volume of Ar^1^, measured as molar refractivity, greatly improves the inhibitory activity of derivatives **2** (IC_50_ = 7.5 μM), **1** (IC_50_ = 51.5 μM), and **6** (IC_50_ = 27.5 μM) in comparison to **3** (43% at 100 μM), **9** (inactive) [27], and **7** (IC_50_ = 35 μM), respectively. The presence of a bulkier substituent Ar^2^ rendered pyrrole **9** inactive compared to the moderate inhibitor **4** (IC_50_ = 100 μM), whereas it increased the anti-LOX activity of hybrid **5** (IC_50_ = 30 μM) in comparison to **8** (IC_50_ = 39 μM). With the exception of **6**, the rest of the hybrids were more potent LOX inhibitors relative to their pyrrole precursors. All hybrids exhibited stronger anti-LOX activity compared to cinnamic acid (Table 2).

The COX isoforms catalyze the dioxygenation (cyclooxygenase activity) and subsequent reduction (peroxidase activity) of arachidonic acid to prostaglandin G_2_ (PGG_2_) and H_2_ (PGH_2_), respectively. The accumulation of any hydroperoxide derived from arachidonic acid or any fatty acid substrate leads to the inactivation of the enzyme. Hence, it is imperative that a co-substrate such as *N*,*N*,*N*′,*N*′-tetramethyl-*p*-phenylenediamine (TMPD) is added to undergo co-oxidation by PGG_2_. The oxidized TMPD has an absorbance maximum at 590 nm. COXs require heme (Fe^3+^-protoporphyrin IX) as a cofactor which may dissociate from the protein during its purification. Therefore, in the in vitro enzymatic assay of ovine COX-2 inhibition, heme is added to ensure maximal enzyme activity [54]. Compounds **1**, **4**, **5**–**8** presented stronger anti-COX-2 activity than indomethacin. With the exception of pyrroles **3** (12% at 100 μM) and **2** (17% at 100 μM), all the other derivatives showed a high inhibitory activity against COX-2. Pyrrole **4** [31] and hybrid **5** were the most potent COX-2 inhibitors with IC_50_ values of 0.65 μM and 0.55 μM, respectively. It seems that lipophilicity and molecular volume do not have any effect on biological activity. The increase in the volume of substituent Ar^1^ only marginally improved the anti-COX-2 activity of **2** (17% at 100 μM) and **6** (IC_50_ = 7.0 μM) in comparison to **3** (12% at 100 μM) and **7** (IC_50_ = 7.2 μM), respectively. The replacement of the phenyl ring with a bulkier substituent Ar^2^ generated hybrid **5** with an enhanced inhibitory activity (IC_50_ value of 0.55 μM) compared to **8** (IC_50_ = 6 μM) [31]. Hybrids **7** (IC_50_ = 7.2 μM) and **6** (IC_50_ = 7.0 μM) were more potent COX-2 inhibitors than their corresponding pyrrole derivatives **3** (12% at 100 μM) and **2** (17% at 100 μM) (Table 2).

The preliminary results indicate that pyrrole **2** presented the highest anti-LOX activity, while pyrroles **1**, **4** and hybrids **5**–**8** could act as potent dual COX-2/S-LOX inhibitors with low-to-moderate antioxidant activity. Derivatives **5** and **6** showed remarkable multifunctional activities among the compounds prepared.

### 2.4. Computational Studies

#### 2.4.1. Docking Simulations on Soybean Lipoxygenase

The synthesized derivatives **2**, **5**, **6**, and **8** were subjected to molecular docking studies on soybean lipoxygenase-1 (PDB ID: 3PZW). They have been studied for their binding mode to the active site and to the whole protein. From the recent literature, it can be revealed that LOXs present—apart from the substrate-binding site (iron-binding site)—potential allosteric binding sites [55,56,57]. All compounds presented allosteric interactions with SLOX-1. It is possible that the large molecular volume of the molecules prevents their access to the active site.

**Figure 3:**
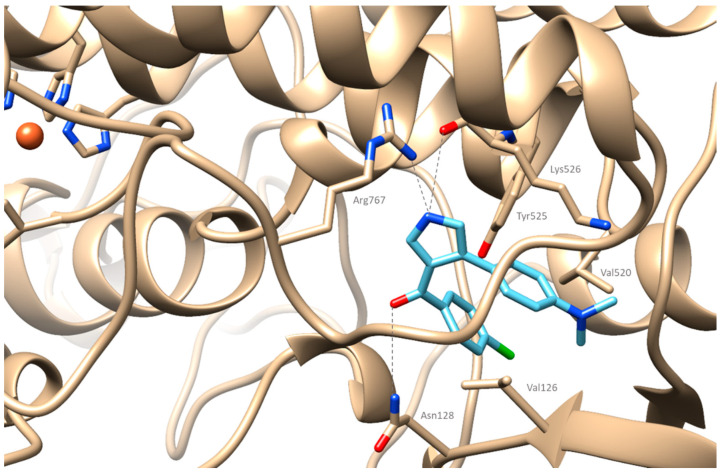
Preferred docking pose of **2** (depicted in cyan) bound to soybean lipoxygenase-1 (SLOX-1). Blue color refers to nitrogen atoms, red refers to oxygen atoms and light green to chlorine while the iron ion is depicted as an orange sphere.

The preferred docking orientation for the most potent compound **2** is shown in Figure 3. Pyrrole **2**, presenting an IC_50_ value of 7.5 μM, had an AutoDock Vina score of −8.6 kcal/mol and seemed to develop (a) hydrophobic interactions with amino acids Val126, Val520, Tyr525, and Lys526; (b) hydrogen bonding interactions with the amino group of Asn128, and with either the carboxyl group of Lys526 or the guanidinium amino group of Arg767, and (c) cation–π interactions with Lys526.

**Figure 4:**
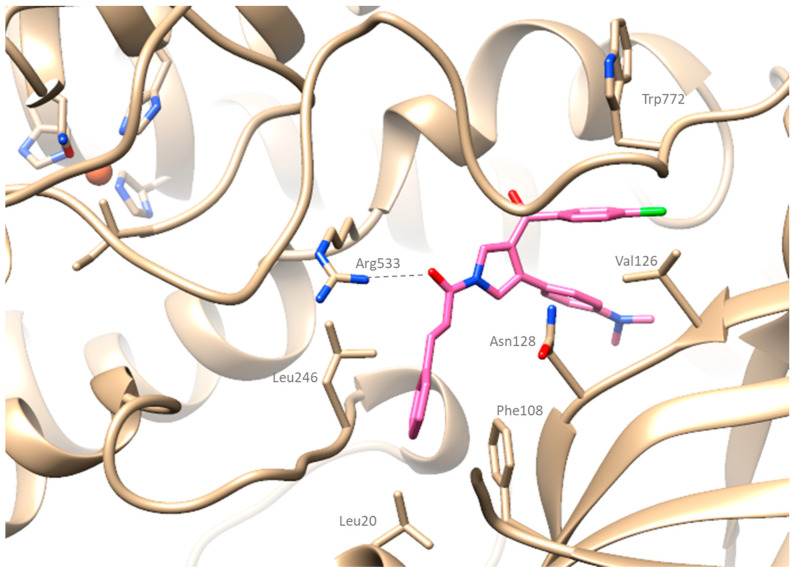
Preferred docking orientation of **6** (depicted in magenta) bound to soybean lipoxygenase-1 (SLOX-1). Blue color refers to nitrogen atoms, red refers to oxygen atoms and light green to chlorine. The iron ion is depicted as an orange sphere.

The most potent hybrid **6** (IC_50_ = 27.5 μM) with an AutoDock Vina score of −10.7 kcal/mol seemed to interact with SLOX-1 through (a) hydrophobic interactions with amino acids Leu20, Val126, Asn128, Leu246, and Trp772; (b) hydrogen bonds between the amidic carbonyl group of **6** and the guanidinium amino group of Arg533; and (c) π-stacking interactions with Phe108, as depicted in Figure 4.

**Figure 5:**
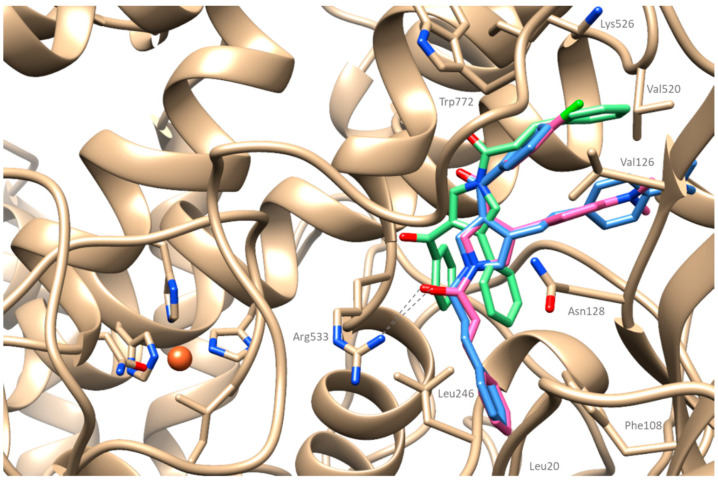
Preferred docking poses of hybrids **6** (magenta), **5** (cornflower blue), and **8** (sea green) bound to soybean lipoxygenase-1 (SLOX-1). Blue color refers to nitrogen atoms, red refers to oxygen atoms and light green to chlorine. The iron ion is depicted as an orange sphere.

We must notice that there was a superposition between hybrids **5** (AutoDock Vina score = −10.9 kcal/mol) and **6**, which presents similar in vitro anti-LOX activities and molecular volume values. Compound **5** seemed to develop (a) hydrophobic interactions with amino acids Leu20, Val126, Asn128, and Trp772; (b) hydrogen bonds between the amidic carbonyl group of **5** and the guanidinium amino group of Arg533; and (c) π-stacking interactions with Phe108. According to the docking orientation of **8** (AutoDock Vina score = −10.3 kcal/mol) illustrated in Figure 5, this hybrid seemed to bind to SLOX-1 through hydrophobic interactions with amino acids Phe108, Val126, Asn128, Leu246, Val520, and Arg533, and cation–π interactions with Lys526.

#### 2.4.2. Docking Simulations on Cyclooxygenase-2

**Figure 6:**
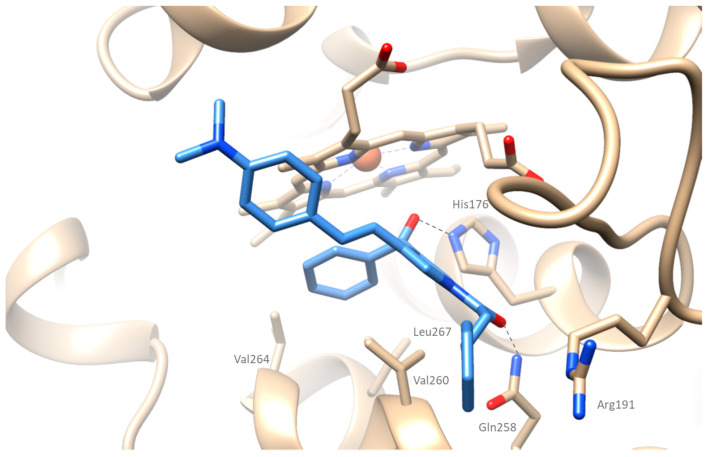
Preferred docking pose of hybrid 5 (depicted in cornflower blue) bound to cyclooxygenase-2 (COX-2). Fe^3+^-protoporphyrin IX is also depicted. Blue color refers to nitrogen atoms and red refers to oxygen atoms. The iron ion is illustrated as an orange sphere.

The most active compounds **5** (IC_50_ = 0.55 μM) presented binding scores of −9.9 kcal/mol. Hybrid **5**, depicted in Figure 6, seemed to develop (a) hydrophobic interactions with Val260, Val264, and Leu267; (b) hydrogen bonds between the carbonyl groups of **5** and the imidazole group of His176 and the amino group of Gln258; and (c) cation–π interactions with Arg19. From our results, it can be concluded that the cinnamic group of hybrid **5** has a great contribution to the molecule’s binding affinity with COX-2 (via hydrogen bonding and π interactions).

## 3. Materials and Methods

### 3.1. General Information

All chemicals, solvents, chemical, and biochemical reagents were of analytical grade and purchased from commercial suppliers (Merck, Merck KGaA, Darmstadt, Germany; Fluka, Sigma-Aldrich Laborchemikalien GmbH, Hannover, Germany; Alfa Aesar, Karlsruhe, Germany and Sigma-Aldrich, St. Louis, MO, USA). All starting materials were obtained from commercial sources and used without further purification. Soybean lipoxygenase, sodium linoleate, 2,2′-azobis(2-methylpropionamidine) dihydrochloride (AAPH), heme, *N*,*N*,*N*′,*N*′-tetramethyl-*p*-phenylenediamine (TMPD), and arachidonic acid were obtained from Sigma Chemical, Co. (St. Louis, MO, USA), while cyclooxygenase-2 (COX-2) was purchased from Cayman.

Melting points were determined on a MEL-Temp II (Laboratory Devices, Holliston, MA, USA) apparatus and were uncorrected. The in vitro tests were performed on a Perkin-Elmer UV/Vis Spectrometer Lamda 20 (Perkin-Elmer Corporation Ltd., Lane Beaconsfield, Bucks, UK) and a Shimadzu UV/Vis Spectrophotometer (UV-1700 Pharmaspec, Shimadzu, Kiyoto, Japan). Infrared (IR) spectra were recorded on a Perkin-Elmer spectrum BX FT-IR spectrometer and the samples were prepared as potassium bromide disks. The nuclear magnetic resonance (NMR) spectra were recorded on an Agilent 500/54 DD2 spectrometer (at 500 MHz for ^1^H-NMR and 126 MHz for ^13^C-NMR, Agilent, Santa Clara, CA, USA) in CDCl_3_ or DMSO-*d*_6_ using tetramethylsilane as an internal standard. Chemical shifts are expressed in units of *δ* (ppm) and coupling constants *J* in Hz. The following abbreviations are used: br = broad, s = singlet, d = doublet, t-triplet, m = multiplet. LC-MS (ESI − MS) spectra were recorded on a Shimadzu LCMS-2010 EV instrument (Shimadzu, Kiyoto, Japan) using methanol as solvent. The elemental analysis for C, H, and N gave values acceptably close to the theoretical values (±0.4%) in a Perkin-Elmer 240B CHN analyzer (Perkin-Elmer Corporation Ltd., Lane Beaconsfield, Bucks, UK).

Reactions were monitored by thin-layer chromatography on 5554 F_254_ Silica gel/TLC cards (Merck and Fluka Chemie GmbH Buchs, Steinheim, Switzerland). The developed TLC plates were subjected to ultraviolet (UV) light or exposed to iodine vapor. Column chromatography was performed using Merck 230–400 mesh silica gel. For preparative thin layer chromatography (PTLC), silica gel 60 F_254_, plates 2 mm, and Merck KGaAICH078057 were used, whereas for the experimental determination of lipophilicity, reversed-phase thin-layer chromatography (RPTLC) 20 × 20 cm TLC-Silica gel 60 F_254_ DC Kieselgel plates (Merck) were utilized.

### 3.2. Chemistry General Procedures

#### 3.2.1. General Procedure for the Synthesis of Pyrroles

The synthesis of pyrrole derivatives **1**–**3** was accomplished through a one-pot reaction, as shown in Scheme 2, according to our previous study [31]. A mixture of a suitable aldehyde (1.0 mmol), enolizable ketone (1.0 mmol), and lithium hydroxide monohydrate (0.1 mmol) in absolute ethanol (3 mL) was stirred at room temperature. The progress of the reaction was monitored by TLC. After the completion of the reaction, *p*-tosylmethyl isocyanide (2.4 mmol) and lithium hydroxide monohydrate (2.2 mmol) were added and the reaction mixture was stirred at room temperature for approximately 24 h. The precipitate was filtered, washed with cold water, and recrystallized from appropriate solvents.

*(E)-(4-chlorophenyl) (4-(4-(dimethylamino)styryl)-1H-pyrrol-3-yl)methanone* (**1**): The aforementioned general method was followed with minor modifications. In the second step, 2.7 mmol of lithium hydroxide monohydrate and 2.4 mmol of *p*-tosylmethyl isocyanide were added. The residue was treated with ethyl acetate and recrystallized to give the pure product. Yield: 39%; *R*_f_: 0.47 (*n*-hexane-ethyl acetate, 2:1, *v*/*v*); decomposes at 229–230 °C; IR (KBr, cm^−1^): 3186 (Ν-H), 1603 (C=O); ^1^H-NMR (500 MHz, DMSO-*d_6_*) δ 11.62 (br s, 1H), 7.75–7.71 (m, 2H), 7.58–7.54 (m, 2H), 7.41 (d, *J* = 16.6 Hz, 1H), 7.30–7.26 (m, 3H), 7.19 (s, 1H), 6.87 (d, *J* = 16.6 Hz, 1H), 6.70 (d, *J* = 8.4 Hz, 2H), 2.90 (s, 6H); ^13^C-NMR (126 MHz, DMSO-*d*_6_) δ 189.8, 149.5, 139.4, 136.0, 130.4, 128.4, 128.3, 126.8, 126.7, 126.1, 123.8, 120.1, 117.5, 117.1, 112.5, 40.1; LC-MS (ESI, *m*/*z*): [Μ + CH_3_OH + Κ]^+^ = 421/423 (3:1), [Μ − N(CH_3_)_2_]^+^· − ·H = 305/307 (3:1), [Μ − H]^1−^ = 349/351 (3:1); elemental analysis calculated: C 71.89, H 5.46, N 7.98 Found: C 71.95, H 5.32, N 7.63.

*(4-chlorophenyl) (4-(4-(dimethylamino) phenyl)-1H-pyrrol-3-yl) methanone* (**2**): The general method was followed. The residue was treated with ethyl acetate and methanol drops and recrystallized to give the pure product. Yield: 18%; *R*_f_: 0.46 (*n*-hexane-ethyl acetate, 1:1, *v*/*v*); m.p.: 195–197 °C; IR (KBr, cm^−1^): 3182 (Ν-H), 1611 (C=O); ^1^H-NMR (500 MHz, DMSO-*d*_6_) δ 11.53 (br s, 1H), 7.71 (d, *J* = 8.3 Hz, 2H), 7.50 (d, *J* = 8.3 Hz, 2H), 7.22–7.18 (m, 3H), 6.94 (s, 1H), 6.63 (d, *J* = 8.6 Hz, 2H), 2.86 (s, 6H); ^13^C-NMR (126 MHz, DMSO-*d*_6_) δ 189.2, 148.9, 138.9, 136.1, 130.8, 129.1, 128.2, 128.0, 125.8, 123.2, 120.1, 118.5, 112.1, 40.3; LC-MS (ESI, *m*/*z*): [Μ + H]^+^ = 325/327 (3:1), [Μ + Na]^+^ = 347/349 (3:1), [Μ + Κ]^+^ = 363/365 (3:1), [Μ + CH_3_OH + Na]^+^ = 379/381 (3:1), [Μ − H]^1−^ = 323/325 (3:1); elemental analysis calculated: C 70.26, H 5.28, N 8.62 found: C 70.0, H 5.35, N 8.78.

*(4-(4-(dimethylamino) phenyl)-1H-pyrrol-3-yl) (thiophen-2-yl) methanone* (**3**): The general method was followed with a few modifications. In the first step, 4-(dimethylamino) benzaldehyde, 2-acetylthiophene, and lithium hydroxide monohydrate were added at a molar ratio of 2:2:1. In the second step, 2.5 mmol of lithium hydroxide monohydrate and 2.5 mmol of *p*-tosylmethyl isocyanide were added. The residue was recrystallized by methanol to give the pure product. Yield: 39%; *R*_f_: 0.71 (*n*-hexane-ethyl acetate, 1:2, *v*/*v*); decomposes at 192–195 °C; IR (KBr, cm^−1^): 3272 (Ν-H), 1609 (C=O); ^1^H-ΝΜR (500 MHz, DMSO-*d*_6_) δ 11.50 (br s, 1H), 7.91 (d, *J* = 4.9 Hz, 1H), 7.69 (d, *J* = 3.6 Hz, 1H), 7.46-7.44 (m, 1H), 7.23–7.18 (m, 3H), 6.95–6.94 (m, 1H), 6.66 (d, *J* = 8.6 Hz, 2H), 2.87 (s, 6H); ^13^C-NMR (126 MHz, DMSO-*d*_6_) δ 181.9, 148.8, 145.9, 133.0, 132.8, 128.8, 128.1, 126.2, 125.4, 123.3, 120.1, 118.2, 112.1, 40.3; LC-MS (ESI, *m*/*z*): [Μ + H]^+^ = 297, [Μ + Na]^+^ = 319, [Μ + Κ]^+^ = 335, [Μ + CH_3_OH + Na]^+^ = 351, [2Μ + Na]^+^ = 615, [Μ − H]^1−^ = 295; elemental analysis calculated: C 68.89, H 5.44, N 9.45 found: C 69.02, H 5.37, N 9.81.

#### 3.2.2. General Procedure for the Synthesis of Pyrrole–Cinnamate Hybrids

Pyrrole–cinnamate hybrids **5**–**7** were prepared according to our previously published procedure [31]. A solution of cinnamoyl chloride (0.7 mmol) in dry dichloromethane (2 mL) was added under argon atmosphere dropwise to a mixture of a suitable pyrrole derivative (1.0 mmol), triethylamine (0.7 mmol), and 4-(dimethylamino) pyridine (0.1 mmol) in dry dichloromethane (4 mL). The reaction mixture was stirred for 24 h at room temperature. Upon completion, the mixture was dissolved in diethylether (50 mL), washed with NaHSO_4_ 10% and NaHCO_3_ 10% solutions, water and brine solution, dried over anhydrous MgSO_4_, filtered, and concentrated under reduced pressure. The final product was purified by either a combination of chromatographic techniques or recrystallization.

*(E)-1-(3-benzoyl-4-((E)-4-(dimethylamino) styryl)-1H-pyrrol-1-yl)-3-phenylprop-2-en-1-one* (**5**): A slightly modified method was applied. Pyrrole **9**, triethylamine, 4-(dimethylamino) pyridine, and cinnamoyl chloride were added at a molar ratio of 1:7.3:0.8:7.4. The final product was isolated by silica gel chromatography (*n*-hexane-ethyl acetate, 5:1, *v*/*v*) and subsequent purification by preparative thin-layer chromatography (*n*-hexane-ethyl acetate, 4:1, *v*/*v*). Yield: 33%; *R*_f_: 0.45 (*n*-hexane-ethyl acetate, 2:1, *v*/*v*); m.p.: 74–76 °C; IR (KBr, cm^−1^): 1698 (C=O)_amide_, 1615 (C=O)_aryl_; ^1^H-NMR (500 MHz, CDCl_3_) δ 8.06 (d, *J* = 15.4 Hz, 1H), 7.89 (d, *J* = 7.5 Hz, 2H), 7.78–7.74 (m, 2H), 7.67–7.65 (m, 2H), 7.62–7.58 (m, 1H), 7.52–7.49 (m, 2H), 7.48–7.45 (m, 3H), 7.39 (d, *J* = 8.6 Hz, 2H), 7.33 (d, *J* = 16.4 Hz, 1H), 7.14 (d, *J* = 15.4 Hz, 1H), 6.99 (d, *J* = 16.4 Hz, 1H), 6.69 (d, *J* = 8.5 Hz, 2H), 2.98 (s, 6H); ^13^C-NMR (126 MHz, CDCl_3_) δ 192.1, 162.7, 150.2, 149.3, 139.6, 134.1, 132.4, 131.6, 130.4, 129.5, 129.3, 128.9, 128.6, 128.4, 127.8, 126.4, 126.0, 125.7, 115.6, 114.6, 113.0, 112.5, 40.6; LC-MS (ESI, *m*/*z*): [Μ−CH_3_]^+·^ = 431, [Μ + Na−2H]^1-^ = 467, [Μ − N(CH_3_)]^+^·− ·H = 401; Elemental analysis calculated: C 80.69, H 5.87, N 6.27 found: C 80.50, H 6.04, N 6.36.

*(E)-1-(3-(4-chlorobenzoyl)-4-(4-(dimethylamino) phenyl)-1H-pyrrol-1-yl)-3-phenylprop-2-en-1-one* (**6**): The general method was followed with a few modifications. Pyrrole **2**, triethylamine, 4-(dimethylamino) pyridine, and cinnamoyl chloride were added at a molar ratio of 1:1.2:0.1:0.9. The final product was purified by two silica gel chromatography systems (*n*-hexane-ethyl acetate, 8:1, *v*/*v*; *n*-hexane-ethyl acetate, 3:1, *v*/*v*). Yield: 25%; *R*_f_: 0.59 (*n*-hexane-ethyl acetate, 3:1, *v*/*v*); m.p.: 180–181 °C; IR (KBr, cm^−1^): 1693 (C=O)_amide_, 1624 (C = O)_aryl_; ^1^H-NMR (500 MHz, CDCl_3_) δ 8.07 (d, *J* = 15.4 Hz, 1H), 7.83–7.79 (m, 3H), 7.67–7.64 (m, 2H), 7.54–7.43 (m, 5H), 7.37 (d, *J* = 8.3 Hz, 2H), 7.25 (s, 1H), 7.15 (d, *J* = 15.4 Hz, 1H), 6.66 (d, *J* = 8.6 Hz, 2H), 2.94 (s, 6H); ^13^C-NMR (126 MHz, CDCl_3_) δ 190.5, 162.8, 150.1, 149.2, 139.0, 137.1, 134.1, 131.6, 131.2, 130.3, 129.4, 129.3, 128.8, 128.7, 126.0, 125.3, 120.9, 117.3, 114.6, 112.4, 40.7; LC-MS (ESI, *m*/*z*): [M + Na]^+^ = 477/479 (3:1); Elemental analysis calculated: C 73.92, H 5.10, N 6.16 found: C 80.03, H 4.88, N 6.32.

*(E)-1-(3-(4-(dimethylamino) phenyl)-4-(thiophene-2-carbonyl)-1H-pyrrol-1-yl)-3-phenylprop-2-en-1-one* (**7**): The general method was followed with a few modifications. Pyrrole **3**, triethylamine, 4-(dimethylamino) pyridine, and cinnamoyl chloride were added at a molar ratio of 1:0.9:0.1:0.8. The residue was treated with ethyl acetate and methanol drops and recrystallized to give the pure product. Yield: 37%; *R*_f_: 0.66 (*n*-hexane-ethyl acetate, 1:1, *v*/*v*); decomposes at 147–148 °C; IR (KBr, cm^−1^): 1694 (C=O)_amide_, 1617 (C=O)_aryl_; ^1^H-ΝΜR (500 MHz, CDCl_3_) δ 8.06 (d, *J* = 15.4 Hz, 1H), 7.96–7.94 (m, 1H), 7.70–7.64 (m, 4H), 7.54–7.52 (m, 1H), 7.48–7.42 (m, 3H), 7.33 (d, *J* = 8.6 Hz, 2H), 7.17 (d, *J* = 15.4 Hz, 1H), 7.10 (t, *J* = 4.3 Hz, 1H), 6.69 (d, *J* = 8.6 Hz, 2H), 2.94 (s, 6H); ^13^C-NMR (126 MHz, CDCl_3_) δ 183.15, 162.80, 150.02, 148.99, 145.24, 134.19, 134.12, 133.91, 131.50, 130.00, 129.29, 129.27, 128.82, 128.08, 126.24, 124.36, 120.98, 117.18, 114.77, 112.49, 40.65; LC-MS (ESI, *m*/*z*): [Μ + Νa]^+^ = 449, [2Μ + Νa]^+^ = 875, [Μ + Νa − 2H]^1−^ = 447; elemental analysis calculated: C 73.21, H 5.20, N 6.57 found: C 72.99, H 5.38, N 6.72.

### 3.3. Physicochemical Studies

#### 3.3.1. Experimental Determination of Lipophilicity as R_M_ Values

Reversed-phase thin-layer chromatography (RPTLC) was used for the determination of R_M_ values. Silica gel plates were saturated with 5% *v*/*v* liquid paraffin in petroleum ether. The mobile phase consisted of a methanol/water mixture (70/30, *v*/*v*). The plates were developed in closed chromatography tanks saturated with the mobile phase. Spots were detected under UV light or after exposure to iodine vapor. R_M_ values were calculated from the corresponding *R*_f_ values (from five individual measurements) using Equation (6) [58].

R_M_ = log[(1/*R*_f_) − 1], (2)

where *R*_f_ is obtained by dividing the distance covered by the sample by the distance covered by the mobile phase.

#### 3.3.2. In Silico Determination of Lipophilicity as ClogP and miLogP Values

Lipophilicity was theoretically calculated as ClogP values by the Bio-Loom program of BioByte Corp. [38] and as miLogP values by the Molinspiration software (https://www.molinspiration.com/cgi-bin/properties, accessed on 2 May 2023) [39].

#### 3.3.3. In Silico Determination of ADMET Properties and Drug-Likeness

All compounds were subjected to in silico evaluation of their drug-likeness and ADMET profile by the Molinspiration software (www.molinspiration.com) [39] and the PreADMET platform (https://preadmet.webservice.bmdrc.org/) [42].

### 3.4. Biological In Vitro Assays

For the in vitro assays, a stock solution (10 mM) of the tested compounds in DMSO was prepared, from which several dilutions were made with the appropriate buffer.

Each experiment was performed at least in triplicate and the standard deviation of absorbance did not exceed 10%.

#### 3.4.1. Inhibition of Linoleic Acid Lipid Peroxidation

In vitro enzymatic assay was conducted as reported in previous studies [30]. 2,2′-Azobis(2-methylpropionamidine) dihydrochloride (AAPH) was used as a controllable source of thermally produced alkylperoxy free radicals. The production of conjugated diene hydroperoxides via the oxidation of sodium linoleate in an aqueous dispersion at 37 °C was monitored at 234 nm. The results were compared to the reference compound, trolox (93%) (Table 2).

#### 3.4.2. Inhibition of Soybean Lipoxygenase In Vitro

An in vitro study was conducted as reported previously [30]. The tested compounds were dissolved in DMSO (100 µM) and incubated at room temperature with sodium linoleate (0.1 mL) as a substrate and 0.2 mL of a soybean lipoxygenase solution in a buffer solution of Tris:HCl (pH 9.00). The conversion of sodium linoleate to 13-hydroperoxylinoleic acid at 234 nm was recorded and compared with the appropriate standard inhibitor NDGA (IC_50_ = 0.45 µM). Several concentrations (seven different solutions from 100 µM to 0.01 µM) were used for the determination of IC_50_ values. The results are given in Table 2.

#### 3.4.3. Inhibition of Ovine Cyclooxygenase-2 (COX-2)

The in vitro COX-2 inhibitory activity was determined using arachidonic acid (AA) as a substrate and *N*,*N*,*N*′,*N*′-tetramethyl-*p*-phenylenediamine (TMPD) as a co-substrate in a buffer solution of Tris:HCl (pH 8.5) [27]. Ten microliters of the DMSO solution of the compound of interest were added in a test tube containing 730 μL of buffer Tris:HCl (pH 8.5), 50 μL of heme, 10 μL of COX-2 and 100 μL of TMPD. The mixture was incubated for 5 min at 37 °C, and after vigorous shaking, the absorbance was measured at 590 nm and 611 nm. Then, 100 μL of AA were added to the same mixture, followed by incubation for 5 min at 37 °C, and after vigorous shaking, the absorbance was measured at 590 nm and 611 nm. Several concentrations (7 different solutions from 100 µM to 0.01 µM) were used for the determination of IC_50_ values. A blank determination was used first to serve as a negative control. The results were compared to the appropriate standard inhibitor indomethacin (Table 2).

### 3.5. Computational Studies

#### 3.5.1. Molecular Docking Studies on Soybean Lipoxygenase

UCSF Chimera (Resource for Biocomputing, Visualization, and Informatics at the University of California, San Francisco, CA, USA) [59] was used for the visualization of the protein (PDB code: 3PZW). Water molecules were removed, missing residues were added with Modeller (10.3) (Departments of Biopharmaceutical Sciences and Pharmaceutical Chemistry, and California Institute for Quantitative Biomedical Research, Mission Bay Byers Hall, University of California San Francisco, San Francisco, CA, USA) [60], hydrogen atoms and AMBER99SB-ILDN charges were added, and the charge on iron was set to +2.0 with no restraint applied to the iron atom and the ligands. Open Babel (3.1.1) was used to generate and minimize ligand 3D coordinates [61] using the MMFF94 force field [62]. Ligand topologies and parameters were generated by ACPYPE (24 December 2021) [63] using AnteChamber (AmberTools 22.10) [64]. Energy minimizations were carried out using the AMBER99SB-ILDN force field [65] with GROMACS (4.6.5) [66]. Docking was performed with AutoDock Vina (1.2.3) (https://vina.scripps.edu/) (accessed on 29 December 2021) [67] by applying a grid box of size 100 Å, 70 Å, and 70 Å in the x, y, and z axes, respectively. The generation of docking input files and the analysis of the docking results were accomplished with UCSF Chimera. Docking was carried out with an exhaustiveness value of 10 and a maximum output of 20 docking modes.

#### 3.5.2. Molecular Docking Studies on Cyclooxygenase-2

Due to a lack of ovine COX-2 in the PDB, used in the in vitro experiments, for the docking studies, the human cyclooxygenase-2 (PDB ID: 1CX2) was selected due to its high homology with the ovine. The alignment of the primary sequences of the two proteins was accomplished using UniProt (www.uniprot.org) (accessed on 1 October 2021) [68] and the results revealed 86.4% homology. The same procedure described above was applied to conduct the molecular docking studies on COX-2. The grid box was a set of sizes 75 Å, 75 Å, and 75 Å in the x, y, and z axes, respectively. The docking calculations were carried out with an exhaustiveness value of 32 and a maximum output of 20 docking poses.

## 4. Conclusions

In the present study, six novel 3,4-disubstituted pyrrole derivatives and pyrrole–cinnamate hybrids were synthesized, assessed with regard to their activity against several biological targets implicated in inflammation, and compared with two derivatives (**4** and **8**) from our previous investigation [31]. All compounds presented low-to-moderate anti-lipid peroxidation activity (31–62%), except for **3** which was inactive under the reported experimental conditions. All hybrids exhibited a stronger antioxidant activity than their corresponding pyrroles and more potent anti-LOX activity compared to cinnamic acid. Moreover, the majority of the hybrids presented improved their activity against S-LOX (with the exception of **6**) and COX-2 (except for **8**) relative to their pyrrole precursors, indicating the biological importance of the incorporation of the cinnamic moiety into the pyrrole scaffold.

Pyrrole **2** was the most potent LOX-inhibitor with an IC_50_ value of 7.5 μM. Compounds **1** and **5**–**7** exhibited dual COX-2/S-LOX inhibitory activity. Hybrids **5** and **6**, in particular, showed the most interesting multitarget biological profile in vitro, and as a consequence, could act as lead compounds for the design of potent anti-inflammatory agents. All synthesized compounds could be described as drug-like. The docking studies of derivatives **2**, **5**, and **6** revealed that allosteric interactions govern the LOX inhibitory binding activity, while docking simulations of **5** on COX-2 supported the biological importance of the presence of the cinnamic moiety in the hybrid.

## Figures and Tables

**Table 1:** Experimentally determined lipophilicity values R_M_. ClogP values, theoretically predicted lipophilicity values by the Bio-Loom program of BioByte Corp. Theoretically predicted lipophilicity values by the Molinspiration software (miLogP) of pyrrole derivatives **1**–**4** and pyrrole–cinnamate hybrids **5**–**8**.

No.	Compounds	R_M_	ClogP	miLogP
**1**	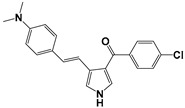	0.3076	5.7	5.32
**2**	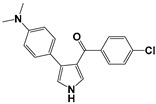	0.0116	5.05	4.74
**3**	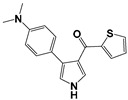	−0.3340	3.98	3.96
**4** [31]	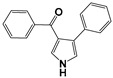	−0.4102	4.08	3.96
**5**	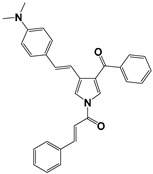	−0.4174	6.68	6.94
**6**	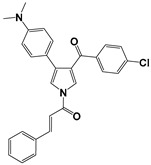	−0.4771	6.87	7.04
**7**	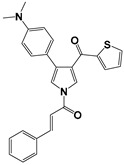	0.6488	5.81	6.26
**8** [31]	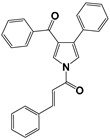	−0.4771	5.85	6.26

**Table 2:** Percent inhibition of lipid peroxidation (ILP % at 100 μM); inhibition of soybean lipoxygenase (LOX Inh. % at 100 μM or IC_50_ μM); inhibition of cyclooxygenase-2 (COX-2 Inh. % at 100 μM or IC_50_ μM); molecular volume calculated by the Molinspiration software (MV); molar refractivity of substituents Ar^1^ and Ar^2^ predicted by the C-QSAR program of BioByte Corp. in previous studies (MR) [27]. The values of the biological experiments are given with their ± SD (standard deviation).

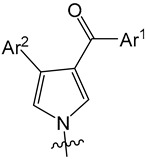						
No.	ILP % at 100 μM	LOX Inh. % at 100 μM or IC_50_ (μM)	COX-2 Inh. % at 100 μM or IC_50_ (μM)	MV	MR (Ar^1^)	MR (Ar^2^)
**1**	33.8 ± 1.3	51.5 ± 1.7 μM	10 ± 0.8 μM	317.69	3.077	4.860
**2**	32.8 ± 0.8	7.5 ± 0.5 μM	17.5% ± 1.1	290.27	3.077	N/A
**3**	no	43.5% ± 1.3	12.5% ± 0.9	267.45	2.395	Ν/A
**4** [31]	44.3 ± 1.1	100 ± 3.3 μM	0.65 ± 0.1 μM	230.83	2.586	2.586
**5**	36.4 ± 0.4	30 ± 1.6 μM	0.55 ± 0.3 μM	422.34	2.586	4.860
**6**	61.6 ± 2.3	27.5 ± 1.3 μM	7.0 ± 0.6 μM	408.46	3.077	N/A
**7**	31.1 ± 1.1	35 ± 1.6 μM	7.2 ± 0.3 μM	385.64	2.395	N/A
**8** [31]	58.1 ± 1.8	39 ± 2.3 μM	6 ± 0.1 μM	349.02	2.586	2.586
**9** [27]	no	no	47% ± 0.7	304.15	2.586	4.860
**Cinnamic acid** [30]	78 ± 3.2	56 ± 1.8 μM	-	-	-	-
**Trolox**	93 ± 2.5	-	-	-	-	-
**NDGA**	-	0.45 ± 0.2μM	-	-	-	-
**Indomethacin**	-	-	2.12 ± 0.6 μM	-	-	-

N/A: not available data.

## Data Availability

The data presented in this study are available in this article.

## Supplementary Materials

The following supporting information can be downloaded at: https://www.mdpi.com/article/10.3390/molecules28247958/s1. Table S1: in silico prediction of drug-likeness and certain absorption, distribution, metabolism, excretion, and toxicity (ADMET) parameters by the Molinspiration software (www.molinspiration.com) and the PreADMET application (https://preadmet.webservice.bmdrc.org/); Figure S1: ^1^H-NMR spectrum of pyrrole **1** in DMSO-*d*_6_, Figure S2: ^13^C-NMR spectrum of pyrrole **1** in DMSO-*d*_6_; Figure S3: ^1^H-NMR spectrum of pyrrole **2** in DMSO-*d*_6_; Figure S4: ^13^C-NMR spectrum of pyrrole **2** in DMSO-*d*_6_; Figure S5: ^1^H-NMR spectrum of pyrrole **3** in DMSO-*d*_6_; Figure S6: ^13^C-NMR spectrum of pyrrole **3** in DMSO-*d*_6_; Figure S7: ^1^H-NMR spectrum of hybrid **5** in CDCl_3_; Figure S8: ^13^C-NMR spectrum of hybrid **5** in CDCl_3_; Figure S9: ^1^H-NMR spectrum of hybrid **6** in CDCl_3_; Figure S10: ^13^C-NMR spectrum of hybrid **6** in CDCl_3_; Figure S11: ^1^H-NMR spectrum of hybrid **7** in CDCl_3_; Figure S12: ^13^C-NMR spectrum of hybrid **7** in CDCl_3_.
