# Supplementary material for: Development of Novel Pyrrole Derivatives and Their Cinnamic Hybrids as Dual COX-2/LOX Inhibitors
Source: Molecules. 2023 Dec 5;28(24):7958. doi: 10.3390/molecules28247958 (PMC10746053; doi:10.3390/molecules28247958)

Table S1. In silico prediction of drug-likeness and certain ADMET (absorption, distribution, metabolism, excretion, toxicity) parameters by the Molinspiration software (www.molinspiration.com) and the PreADMET application (https://preadmet.webservice.bmdrc.org/).

ADMET parameters \ Compound	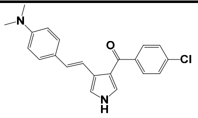 **1**	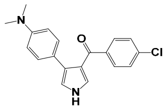 **2**	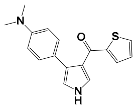 **3**	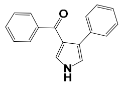 **4***
logBB	0.41336	0.3252	0.20664	0.254592
BBB penetration	High absorption to CNS	High absorption to CNS	High absorption to CNS	High absorption to CNS
CMC like rule	Qualified	Qualified	Qualified	Qualified
Lead like rule	Violated	Violated	Violated	Violated
MDDR like rule	Mid-structure	Mid-structure	Mid-structure	Mid-structure
Rule of Five	Suitable	Suitable	Suitable	Suitable
WDI like rule	Out of 90% cutoff	In 90% cutoff	In 90% cutoff	In 90% cutoff
Caco2 cell permeability	Middle	Middle	Middle	Middle
Human Intestinal Absorption	Well absorbed	Well absorbed	Well absorbed	Well absorbed
MDCK cell permeability	Low	Low	Low	Middle
CYP2C19 inhibition	×	×	×	✓
CYP2C9 inhibition	×	×	×	✓
CYP2D6 inhibition	×	×	×	×
CYP2D6 substrate	×	×	×	×
CYP3A4 inhibition	×	×	×	✓
CYP3A4 substrate	Weakly	Weakly	Weakly	×
P-glycoprotein inhibition	✓	✓	✓	×
Plasma Protein Binding	Strongly bound	Strongly bound	Weakly bound	Strongly bound
Carcinogenicity (mouse)	×	×	×	×
Carcinogenicity (rat)	✓	✓	✓	✓
Ames test in TA100_10RLI	Non-mutagenic	Non-mutagenic	Non-mutagenic	Non-mutagenic
Ames test in TA100_NA	Non-mutagenic	Non-mutagenic	Mutagenic	Mutagenic
Ames test in TA1535_10RLI	Mutagenic	Mutagenic	Mutagenic	Mutagenic
Ames test in TA1535_NA	Non-mutagenic	Mutagenic	Mutagenic	Mutagenic
hERG inhibition	Medium risk	Medium risk	Medium risk	Medium risk
QSAR (2) IC_{50} (nM)	1.5374	3.6957	15.6574	13.6811
QSAR (3) IC_{50} (nM)	7.8181	20.5897	101.3771	87.3454
QSAR (4) IC_{50} (nM)	1.902×10^{-9}	3.816×10^{-5}	2.1033	1.0073
QSAR (5) IC_{50} (μM)	8.3138	3.4174	0.79090	0.9068

ADMET parameters \ Compound	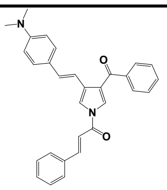<br>**5**	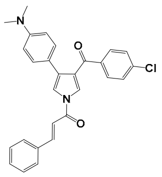<br>**6**	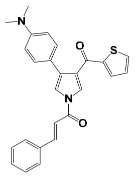<br>**7**	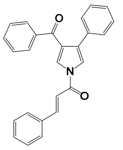<br>**8***
logBB	0.567692	0.582892	0.464332	0.512136
BBB penetration	Middle absorption to CNS	Middle absorption to CNS	Middle absorption to CNS	Middle absorption to CNS
CMC like rule	Not qualified	Not qualified	Not qualified	Not qualified
Lead like rule	Violated	Violated	Violated	Violated
MDDR like rule	Drug-like	Mid-structure	Mid-structure	Mid-structure
Rule of Five	Suitable	Suitable	Suitable	Suitable
WDI like rule	Out of 90% cutoff	Out of 90% cutoff	Out of 90% cutoff	Out of 90% cutoff
Caco2 cell permeability	Middle	Middle	Middle	Middle
Human Intestinal Absorption	Well absorbed	Well absorbed	Well absorbed	Well absorbed
MDCK cell permeability	Low	Low	Low	Middle
CYP2C19 inhibition	×	×	×	✓
CYP2C9 inhibition	✓	✓	✓	✓
CYP2D6 inhibition	×	×	×	×
CYP2D6 substrate	×	×	×	×
CYP3A4 inhibition	×	×	×	✓
CYP3A4 substrate	Weakly	Weakly	Weakly	Weakly
P-glycoprotein inhibition	✓	✓	✓	✓
Plasma Protein Binding	Strongly bound	Strongly bound	Strongly bound	Strongly bound
Carcinogenicity (mouse)	✓	✓	✓	✓
Carcinogenicity (rat)	×	✓	✓	×
Ames test in TA100_10RLI	Non-mutagenic	Non-mutagenic	Non-mutagenic	Mutagenic
Ames test in TA100_NA	Non-mutagenic	Non-mutagenic	Non-mutagenic	Non-mutagenic
Ames test in TA1535_10RLI	Mutagenic	Mutagenic	Mutagenic	Mutagenic
Ames test in TA1535_NA	Non-mutagenic	Non-mutagenic	Non-mutagenic	Non-mutagenic
hERG inhibition	Medium risk	Medium risk	Medium risk	Medium risk
QSAR (2) IC_{50} (nM)	0.4097	0.3171	1.3254	1.2557
QSAR (3) IC_{50} (nM)	1.8157	1.3681	6.6364	6.2524
QSAR (4) IC_{50} (nM)	5.7638×10^{-18}	6.7049×10^{-20}	2.7848×10^{-10}	1.3606×10^{-10}
QSAR (5) IC_{50} (μM)	31.7629	41.1889	9.6636	10.2070

*The indicated in **Table S1** in silico results for compounds **4** and **8** are not included in publication [31]

Figure S1. ^{1}H-NMR spectrum of pyrrole **1** in DMSO-d_6.

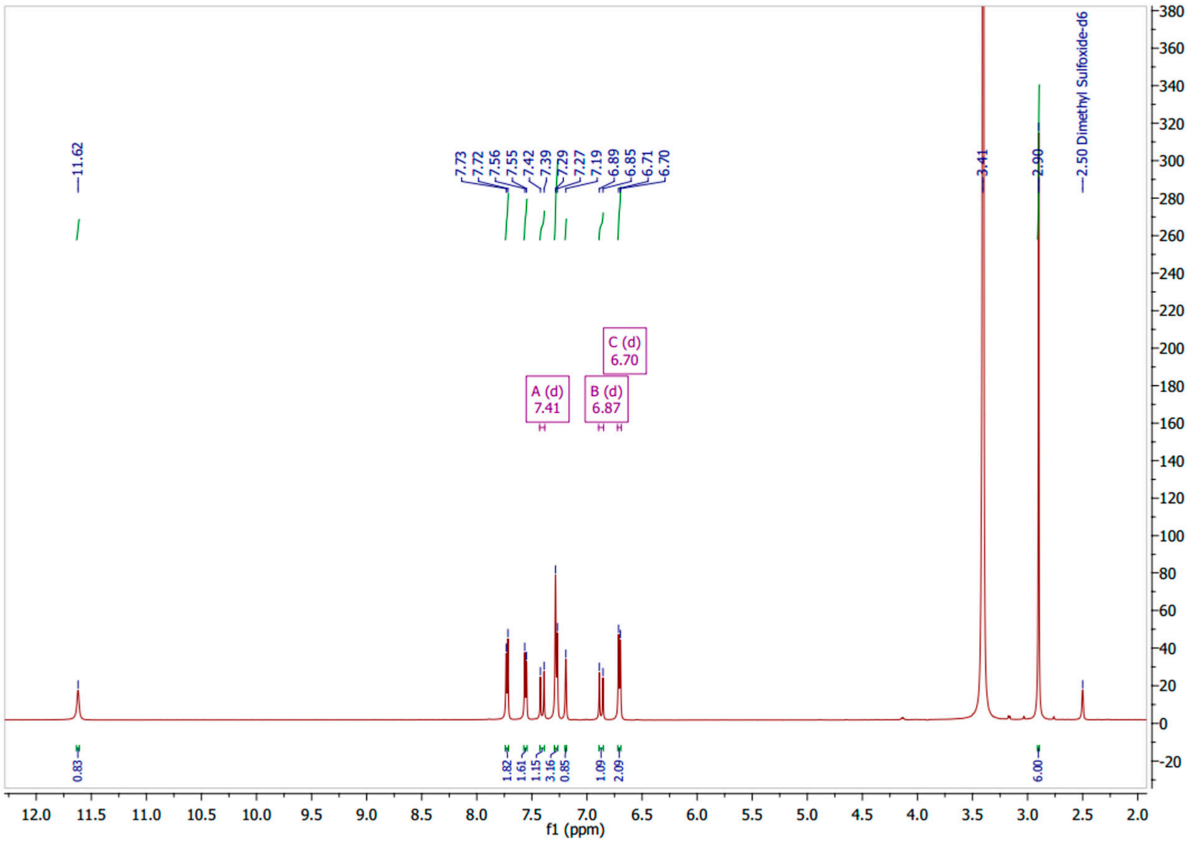

Figure S2. ^{13}C-NMR spectrum of pyrrole **1** in DMSO-d_6.

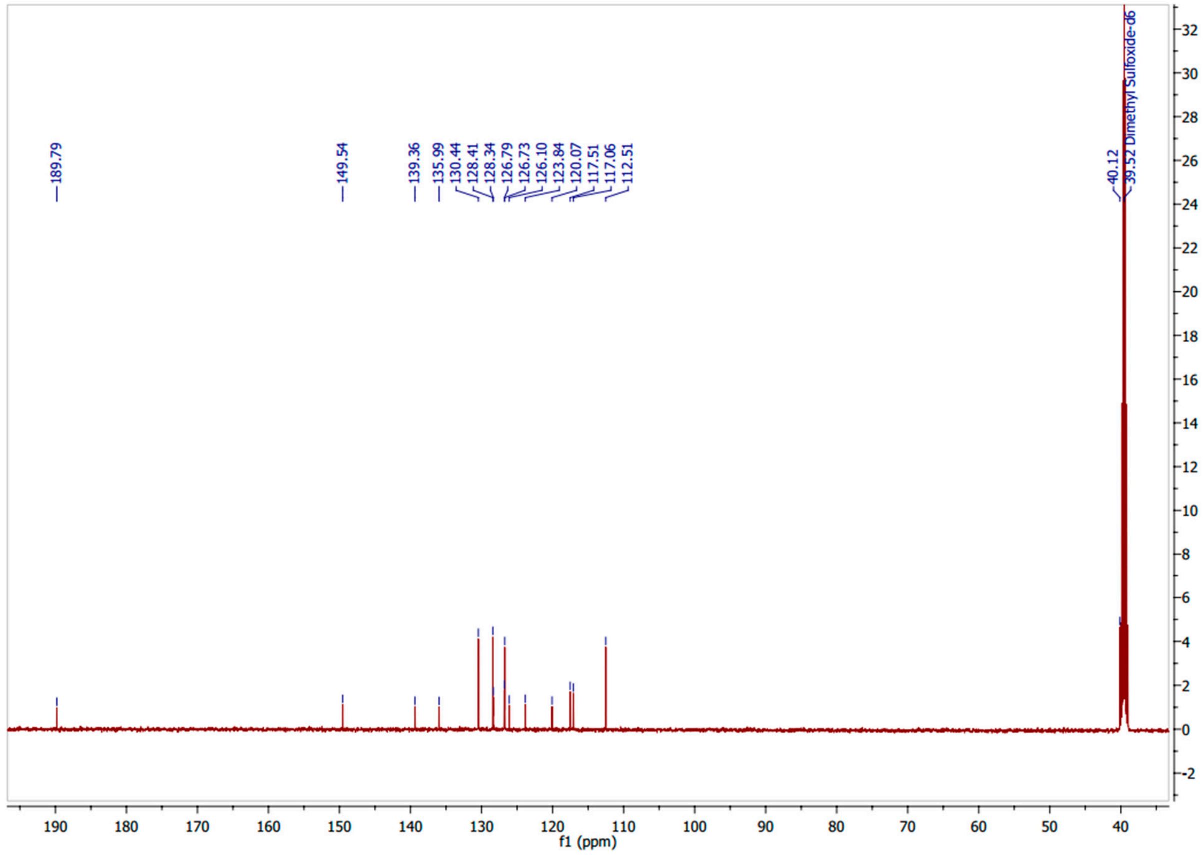

Figure S3. ^{1}H-NMR spectrum of pyrrole **2** in DMSO-d_6.

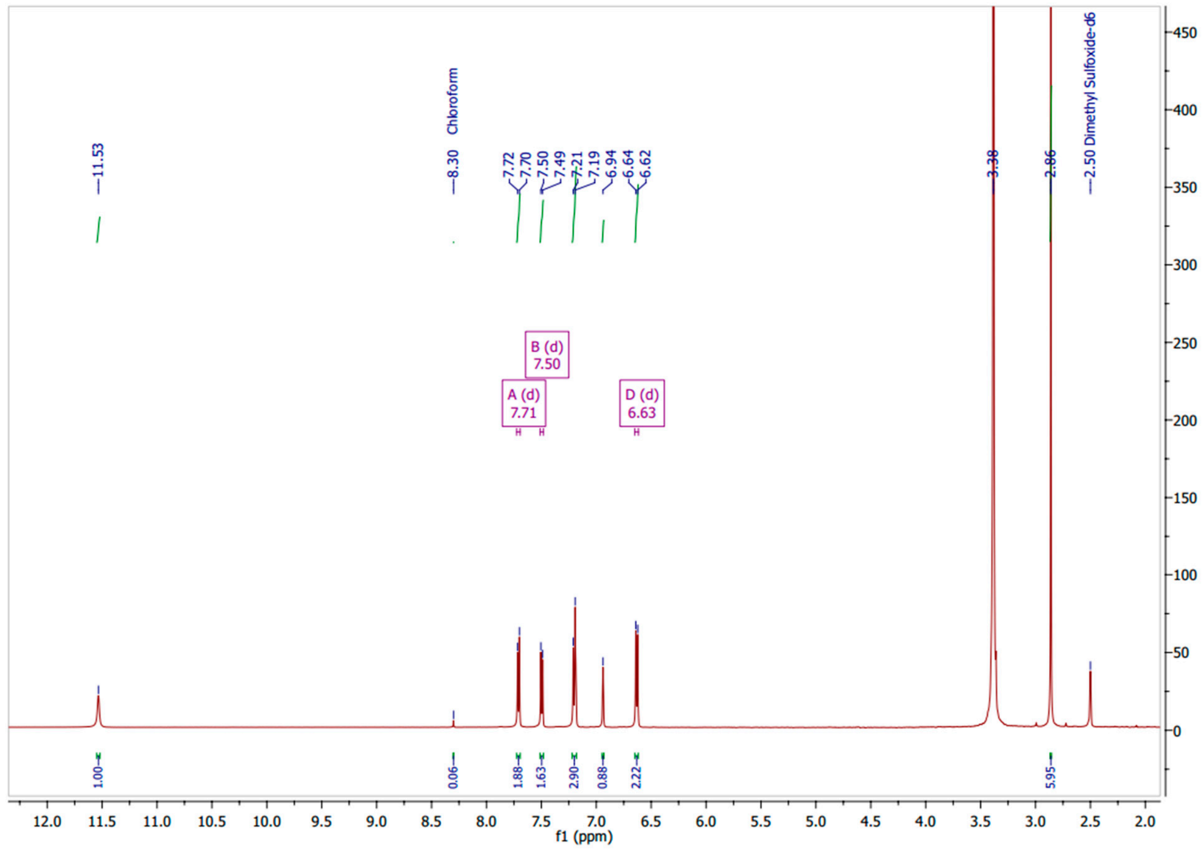

Figure S4. ^{13}C-NMR spectrum of pyrrole **2** in DMSO-d_6.

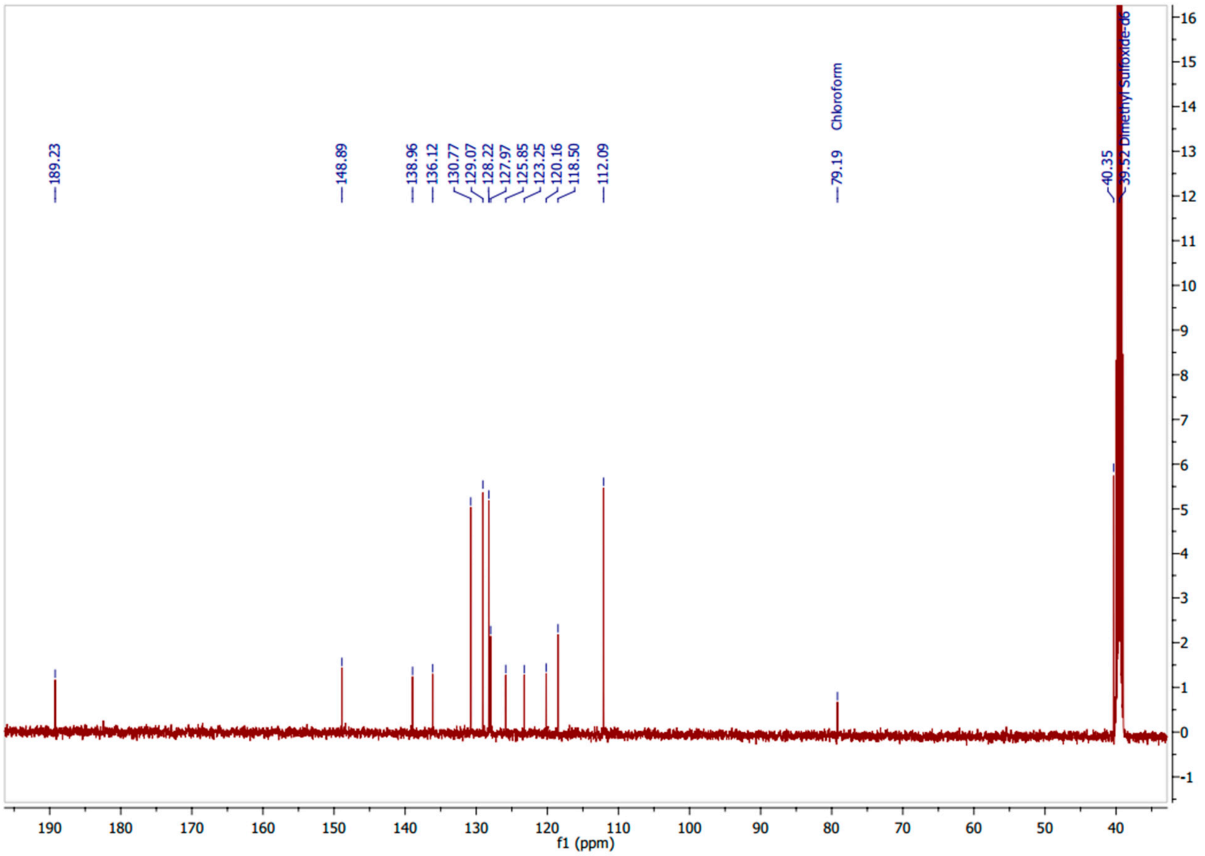

Figure S5. ^{1}H-NMR spectrum of pyrrole **3** in DMSO-d_6.

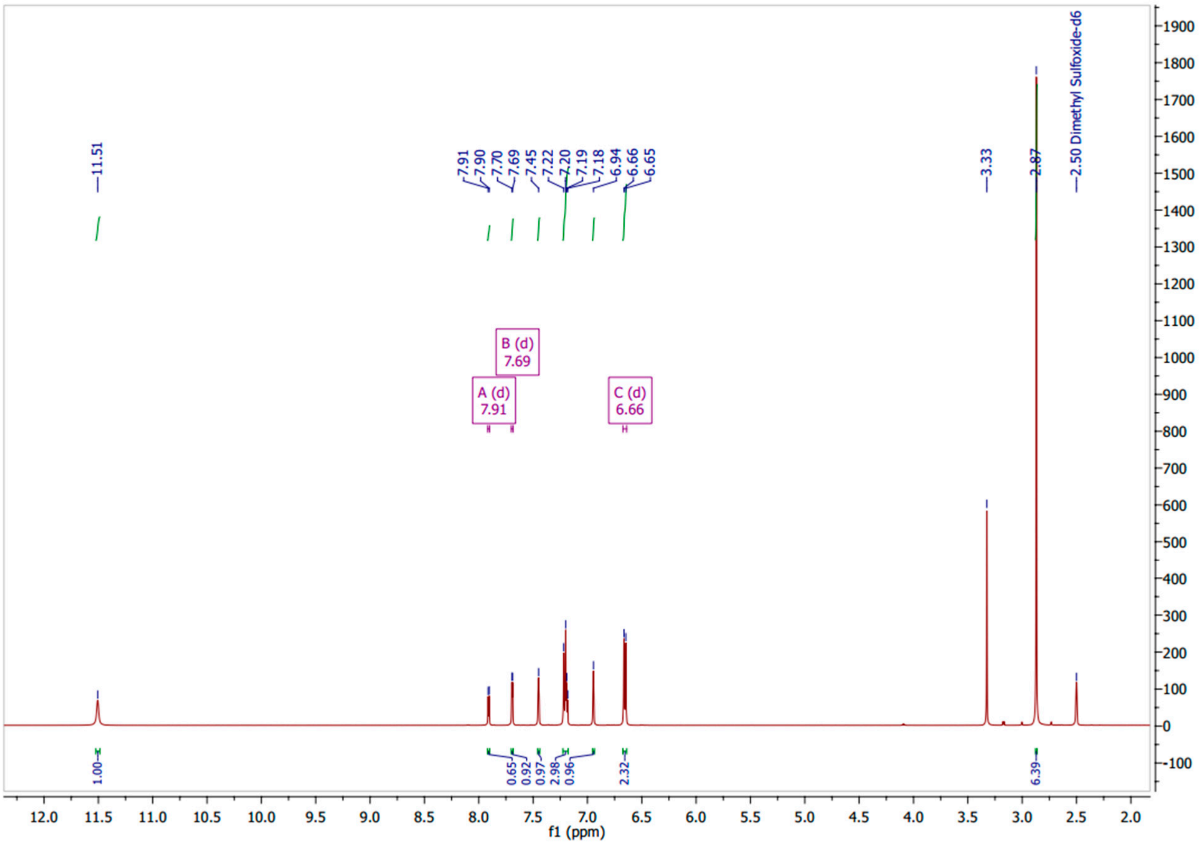

Figure S6. ^{13}C-NMR spectrum of pyrrole **3** in DMSO-d_6.

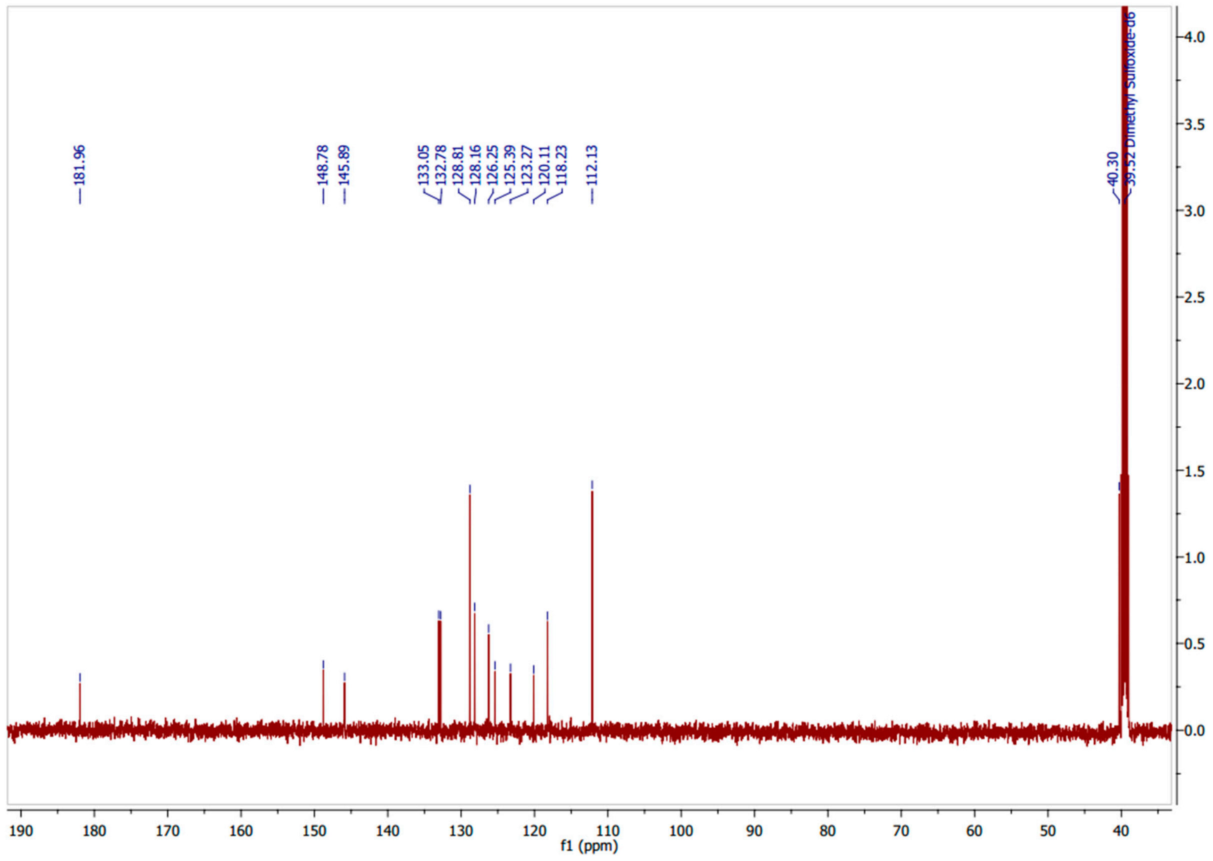

Figure S7. ^{1}H-NMR spectrum of hybrid **5** in $CDCl_3$.

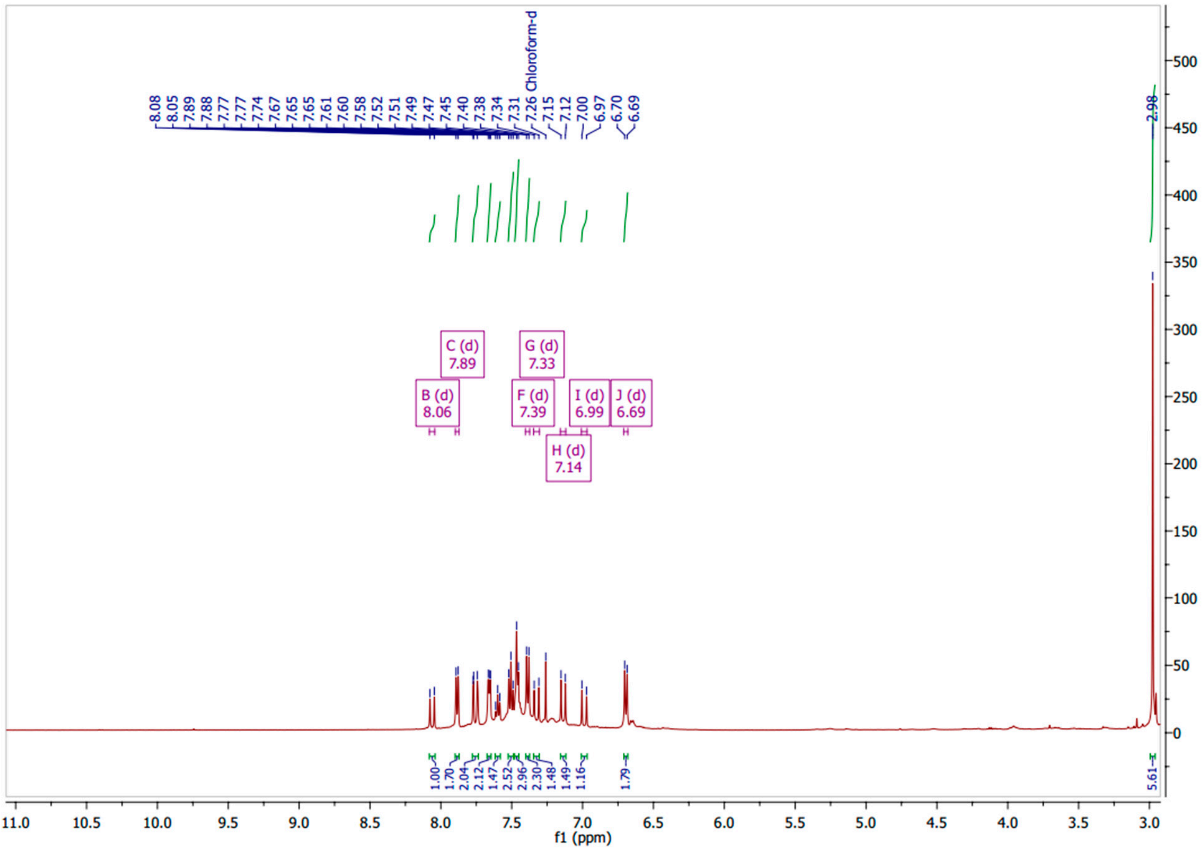

Figure S8. ^{13}C-NMR spectrum of hybrid **5** in $CDCl_3$.

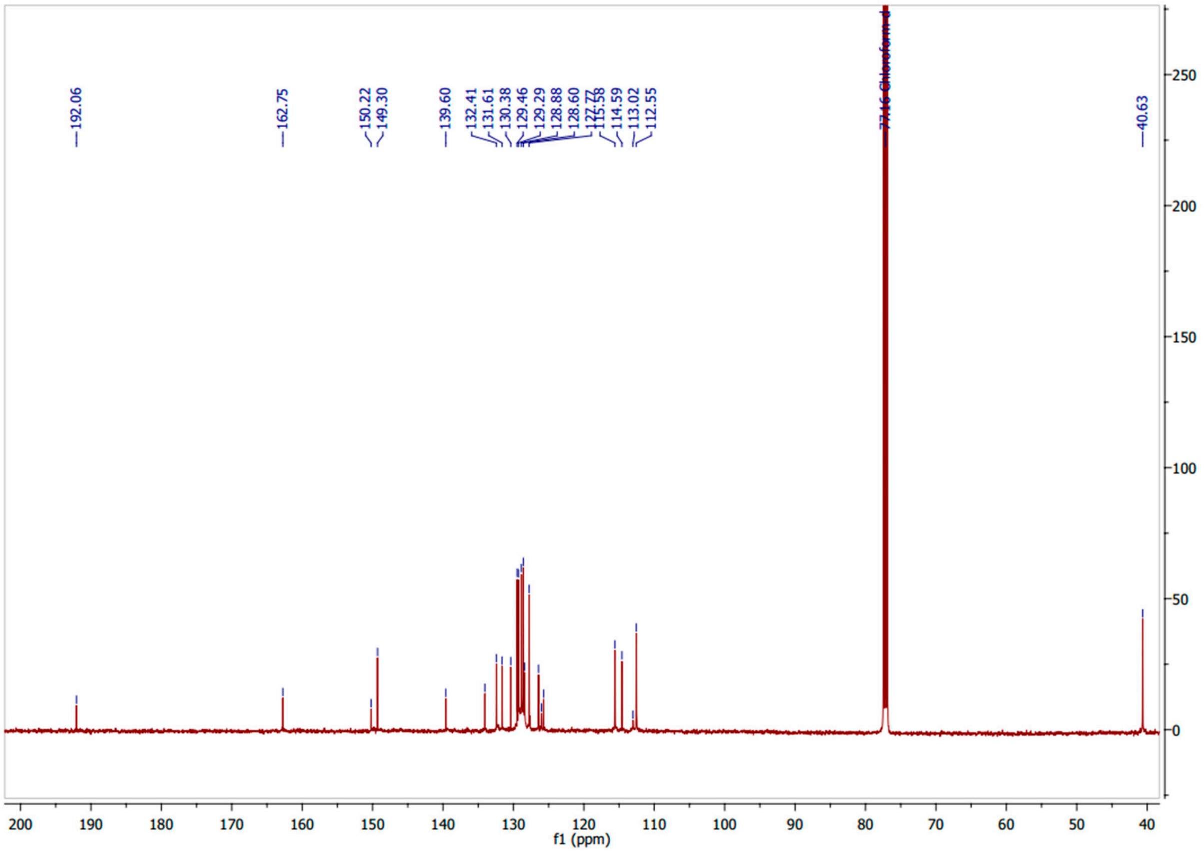

Figure S9. ^{1}H-NMR spectrum of hybrid **6** in $CDCl_3$.

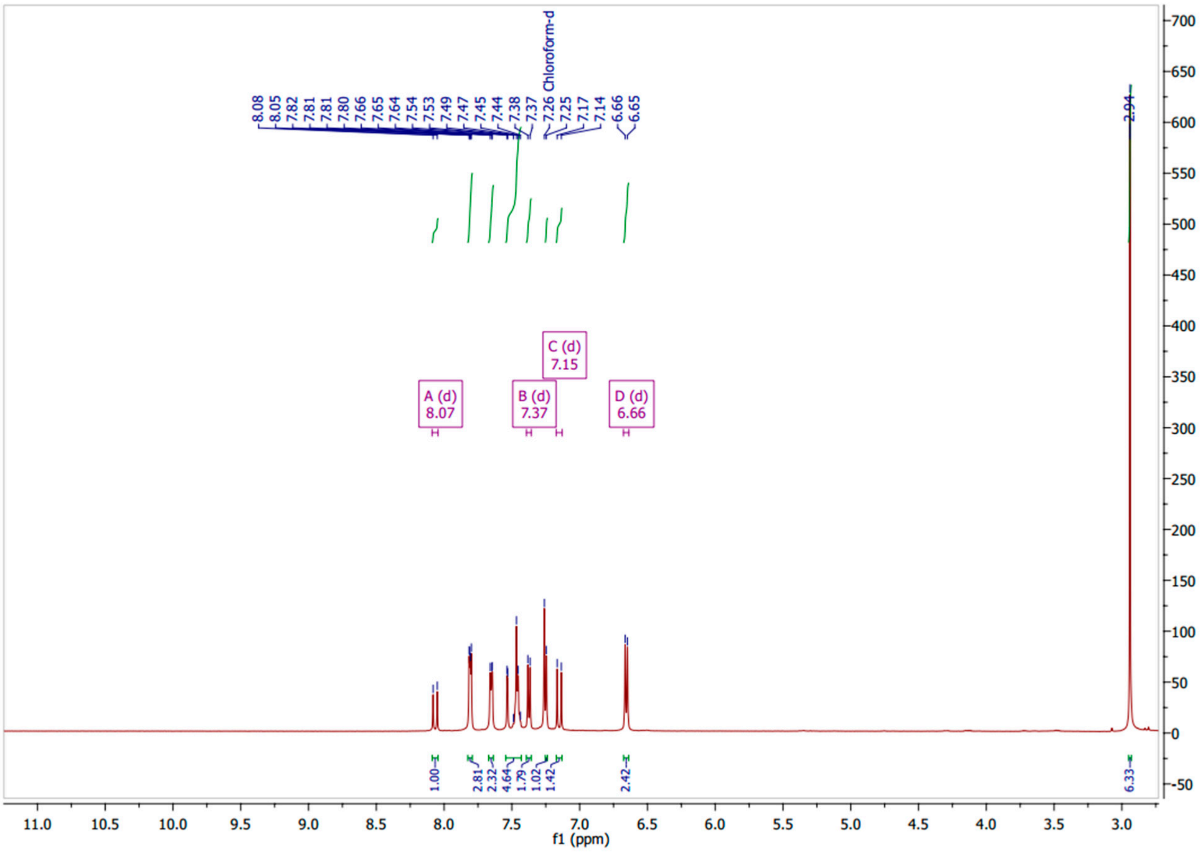

Figure S10. ^{13}C-NMR spectrum of hybrid **6** in $CDCl_3$.

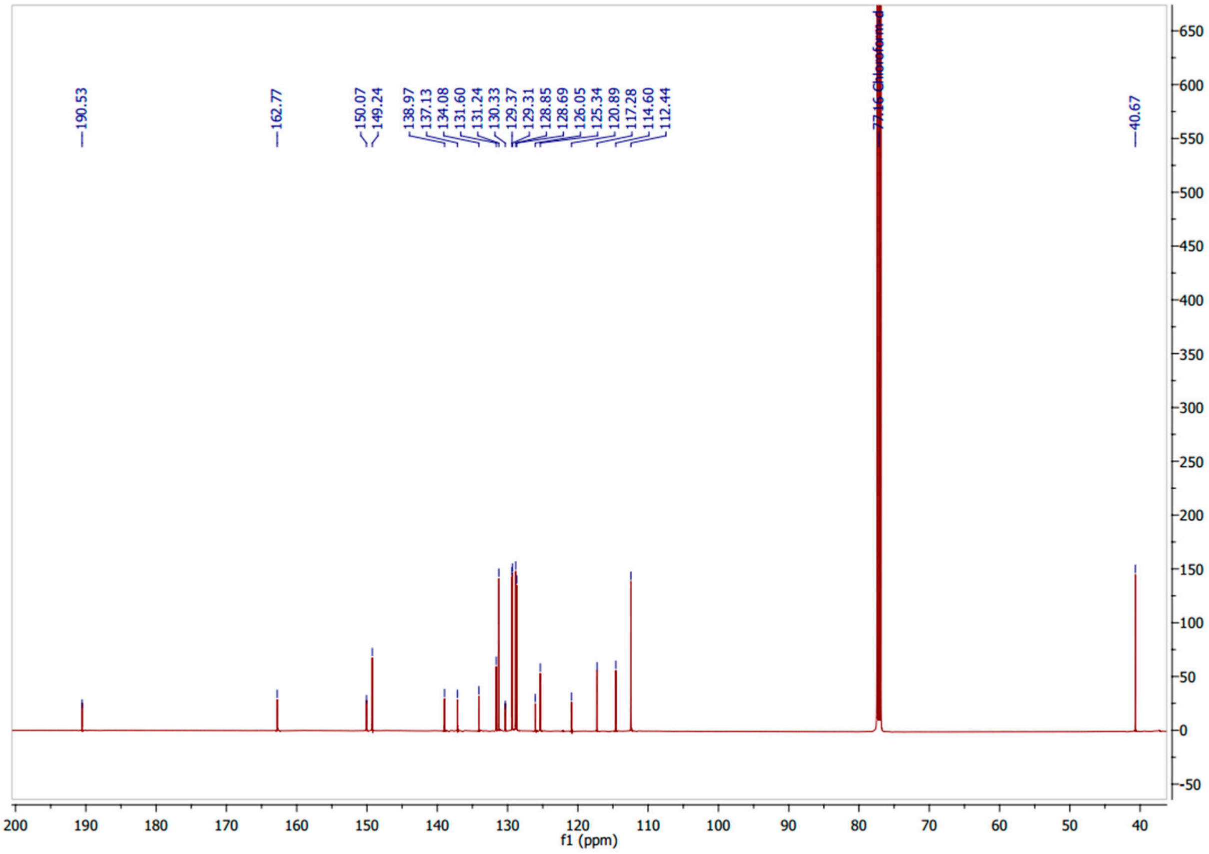

Figure S11. ^{1}H-NMR spectrum of hybrid **7** in $CDCl_3$.

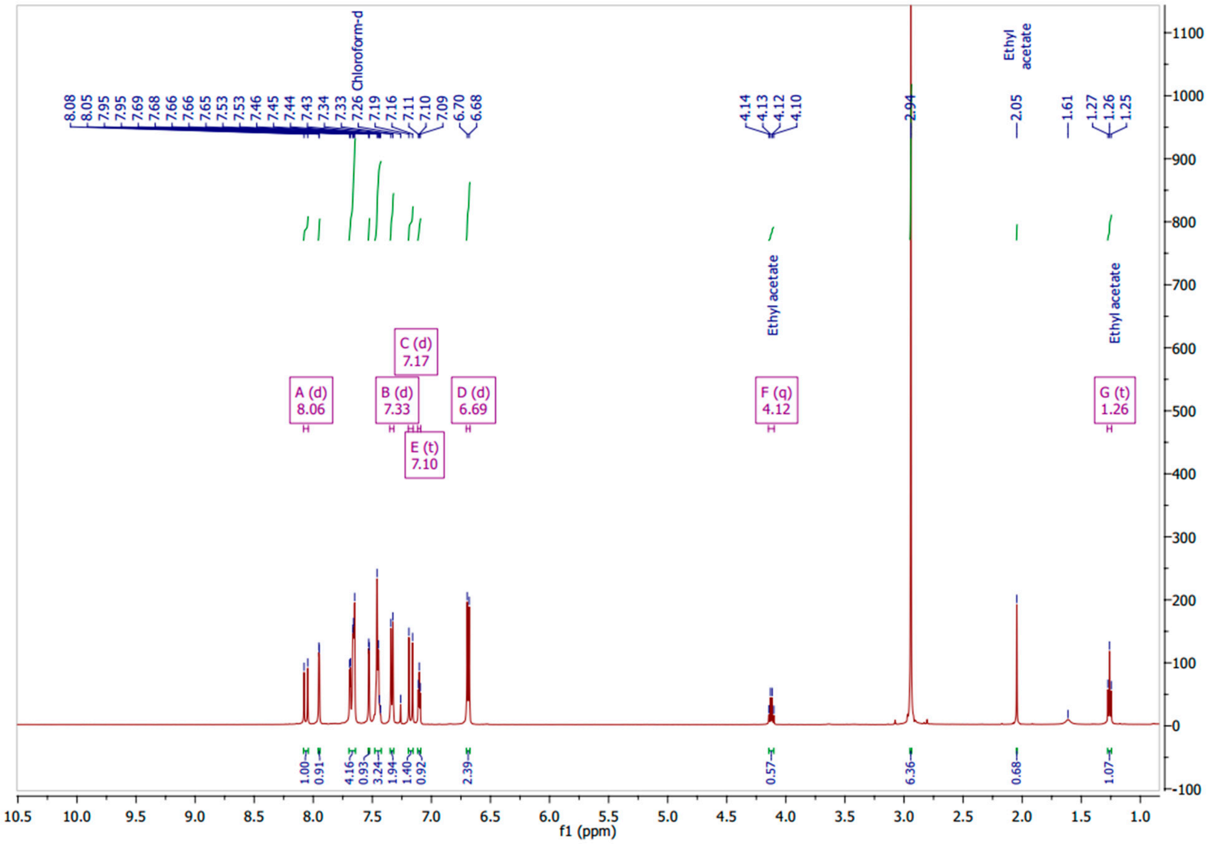

Figure S12. ^{13}C-NMR spectrum of hybrid **7** in $CDCl_3$.

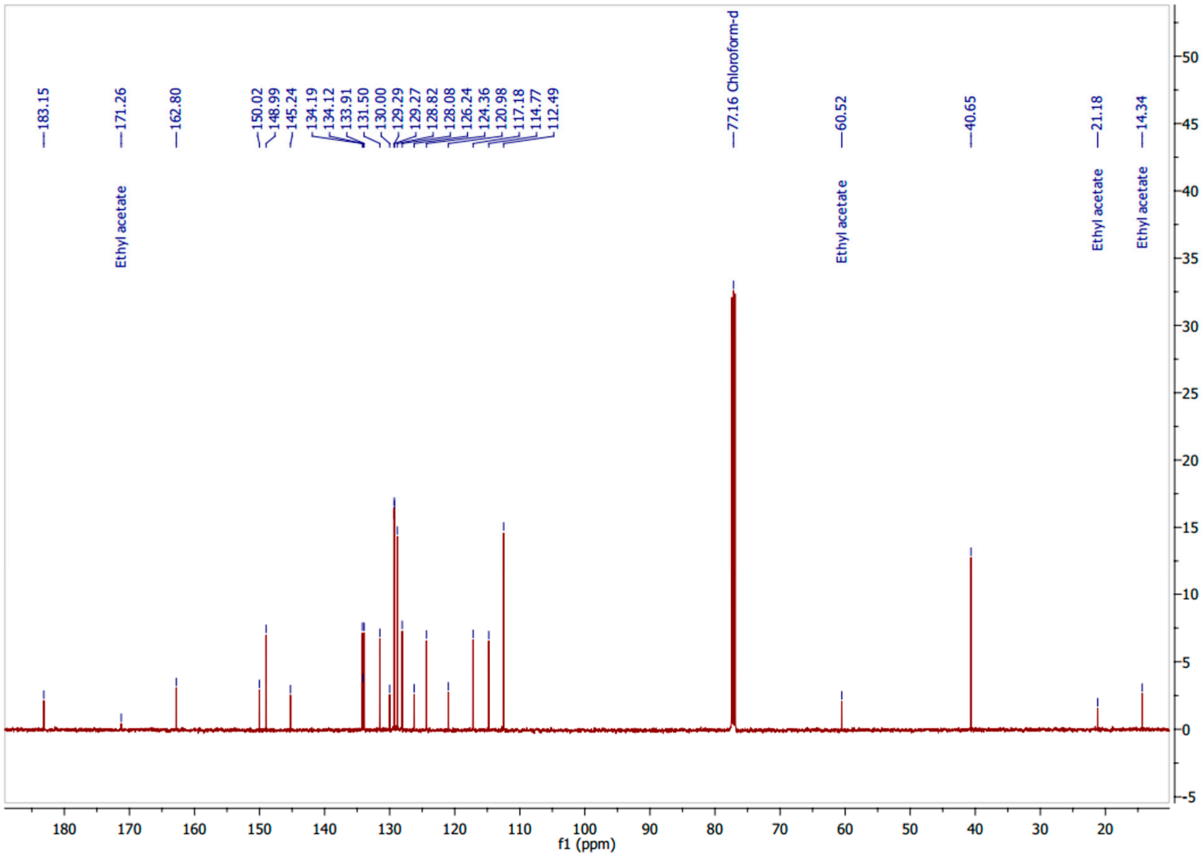

Supplement: Supplementary file 1 [file molecules-28-07958-s001.zip › molecules-2628284-supplementary.pdf]
